# Couple serostatus patterns in sub-Saharan Africa illuminate the relative roles of transmission rates and sexual network characteristics in HIV epidemiology

**DOI:** 10.1038/s41598-018-24249-7

**Published:** 2018-04-27

**Authors:** Steven E. Bellan, David Champredon, Jonathan Dushoff, Lauren Ancel Meyers

**Affiliations:** 1 Department of Epidemiology and Biostatistics, College of Public Health, University of Georgia, Athens, Georgia United States of America; 2 Center for Ecology of Infectious Diseases, University of Georgia, Athens, Georgia United States of America; 3 Department of Biology, McMaster University, Hamilton, Ontario, Canada; 4 Department of Integrative Biology, University of Texas at Austin, Austin, Texas United States of America; 5 The Santa Fe Institute, Santa Fe, New Mexico United States of America

## Abstract

HIV prevalence has surpassed 30% in some African countries while peaking at less than 1% in others. The extent to which this variation is driven by biological factors influencing the HIV transmission rate or by variation in sexual network characteristics remains widely debated. Here, we leverage couple serostatus patterns to address this question. HIV prevalence is strongly correlated with couple serostatus patterns across the continent; in particular, high prevalence countries tend to have a lower ratio of serodiscordancy to concordant positivity. To investigate the drivers of this continental pattern, we fit an HIV transmission model to Demographic and Health Survey data from 45,041 cohabiting couples in 25 countries. In doing so, we estimated country-specific HIV transmission rates and sexual network characteristics reflective of pre-couple and extra-couple sexual contact patterns. We found that variation in the transmission rate could parsimoniously explain between-country variation in both couple serostatus patterns and prevalence. In contrast, between-country variation in pre-couple or extra-couple sexual contact rates could not explain the observed patterns. Sensitivity analyses suggest that future work should examine the robustness of this result to between-country variation in how heterogeneous infection risk is within a country, or to assortativity, i.e. the extent to which individuals at higher risk are likely to partner with each other.

## Introduction

HIV epidemic severity varies extensively across sub-Saharan Africa (SSA), where prevalence has peaked at under 1% in some countries and surpassed 30% in others^1^. Studies examining the cause of this between-country variation generally fall into one of two camps^2,3^. The first proposes a central role of biological cofactors which affect HIV infectivity (circumcision, STI or other coinfections, viral load, pathogen or host genetics)^4–8^, while the second focuses on sexual network characteristics (in particular, the prevalence of concurrent partnerships, but also partner turnover rates, migration, and age-disparate relationships)^9–15^. Cohort and intervention studies have demonstrated a clear relationship between biological cofactors and transmission efficiency^16,17^ but, because of their limited geographic expanse, are insufficient for understanding how geographic differences in these cofactors drive regional variation in epidemic prevalence. Ecologic studies modeling correlations between known cofactors and prevalence are useful for hypothesis generation, but are highly susceptible to confounding^18,19^. Similarly, mathematical modeling studies have shown that certain sexual network characteristics (particularly concurrency) should exacerbate HIV epidemics. But ecologic studies examining whether these same network characteristics drive differences between countries provide mixed results, possibly because they suffer from susceptibility to confounding and from methodological difficulties associated with measuring sexual-network characteristics^19–28^.

Most researchers agree that both biological cofactors and sexual network structure *can* affect HIV transmission dynamics. Nevertheless, after decades of active debate, it remains unclear what *actually* drives the large observed differences between countries’ epidemic trajectories. This debate has practical relevance because the various explanations for what drives between-country variation in HIV prevalence may have important implications for intervention implementation and effectiveness.

We investigated drivers of between-country variation in epidemic severity in SSA by using couple serostatus data to disentangle the components of transmission. Couple serostatus patterns vary widely across SSA. This can be most clearly seen by examining summary indices, such as the serodiscordant proportion (SDP; the proportion of couples with at least one HIV seropositive partner that are serodiscordant). SDP varies considerably across SSA, ranging from 37% to 85%, in inverse correlation with prevalence^8,29,30^. There is a common perception that these SDPs are larger than expected^31,32^, given the intuitive argument that intercourse between stable serodiscordant partners should rapidly produce positive concordance. Heuristic arguments have attributed large SDPs to high extramarital transmission^29,31,32^, heterogeneity in HIV infectiousness or susceptibility^8,29,33–35^, population-level HIV prevalence^29^, and AIDS-related mortality^36^. However, the relative impacts of these factors on SDP remain poorly understood^29^.

Here, we fit mechanistic models of HIV transmission to Demographic and Health Survey (DHS) data on stable cohabiting couples to investigate what processes could explain between-country variation in HIV prevalence and couple serostatus patterns. We evaluate common explanations for between-country variation in epidemic prevalence and exclude those that are inconsistent with individual-level data. The models we are able to fit assume homogeneity, i.e. that, given exposure to an infected individual, all individuals exhibit the same risk of infection. However, we used simulation-based sensitivity analyses to investigate the robustness of our conclusions to heterogeneity in the risk of infection and assortativity therein (whether individuals with higher risk of infection are more likely to form partnerships with each other).

## Methods

The appendix provides a complete model description and all material needed to reproduce our analyses. All analyses were performed in R^37^. First, we fit models to DHS data to estimate transmission rates and sexual contact coefficients for each country’s data. Second, we conducted a meta-analysis of parameter estimates from each country to understand which processes could explain between-country variation in peak HIV prevalence. Third, we conducted sensitivity analyses to explore how these patterns are affected by processes that both were and were not included in our fitted model.

### Formulation of the Couples Transmission Model

We investigated the processes governing the relationship between couple serostatus patterns and HIV prevalence by fitting a model of HIV transmission in stable, cohabiting couples to DHS data for 2003–2012, from 45,041 couples for all 25 countries in SSA for which the DHS included HIV testing (Tables S1–S2). This model extends our previously described framework for analyzing cross-sectional couples data^38^. Generally, unlike cohort data, cross-sectional data cannot be analyzed via survival-like analyses because individuals are only sampled once. In this framework, we are able to analyze cross-sectional survey data as cohort data because, by assuming that surveyed individuals were seronegative at their sexual debut, we have two observation time points per individual. The model used to analyze these data is analogous to a survival analysis for infection data with couples (versus individuals) as the unit of analysis. This model combines characteristics of survival analysis with those from mathematical transmission modeling.

**Figure 1:**
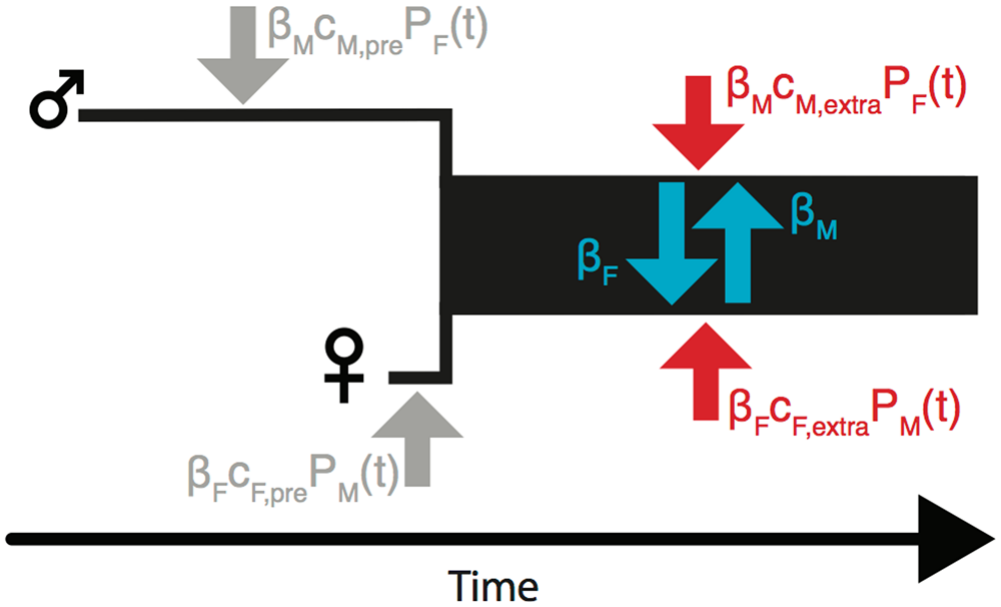
(**A**) Schematic diagram of the mechanistic couples transmission model that we fit to individual-level couples relationship history and serostatus data. During a couple’s relationship (black rectangle), infected individuals infect each other (blue) at gender-specific transmission rates (${\beta }_{M},{\beta }_{F}$); during this time individuals are also infected by extra-couple partners (red) at a rate equal to the product of the transmission rate, the population HIV prevalence (*P*_*M*_(*t*), *P*_*F*_(*t*)), and a sexual contact coefficient (${c}_{M,e},{c}_{F,e}$). Similarly, individuals can be infected by pre-couple partners (gray) starting from their sexual debut up until they form into a couple. The end of the black rectangle denotes the DHS sample date (i.e. when couple serostatus is observed). For *P*_*M*_(*t*) and *P*_*F*_(*t*), we use UNAIDS HIV gender-specific prevalence trajectories and also account for the scale up of ART such that these represent the probability a non-stable partner is both infected and infectious. Using Bayesian MCMC, we estimated the transmission rates and contact coefficients that yielded the highest probability of all couples exhibiting their observed serostatus given their relationship history.

Specifically, we allowed for three hazard rates for each gender, corresponding to pre-couple, extra-couple, and within-couple routes of infection. As illustrated in Fig. 1, we denote $\beta$ as the rate of transmission from the infected to the uninfected partner of a serodiscordant couple (within-couple transmission). The rate at which individuals are infected by casual partners (i.e. either pre-couple or extra-couple partners) is a product of three factors: the transmission rate, a contact rate, and the probability a contact is infectious. The first factor is $\beta$; this is analogous to the use of transmission rates estimated from serodiscordant couple cohort studies^39^ as proxies for a more general HIV transmission rate. The second factor is the rate at which individuals engage in sex with casual partners, $c$. The third factor is the probability that their sexual partners are infectious, *P*(*t*), which denotes the time-varying infectious HIV prevalence (proportion infected and not on antiretroviral therapy (ART) as estimated by UNAIDS [1]) in the opposite sex’s population. As denoted by the subscripts in Fig. 1, we estimate gender-specific parameters and we estimate different contact coefficients, $c$, for pre- and extra-couple transmission routes. This structure allows us to separately estimate gender-specific transmission rates ($\beta$) underlying all routes of infection and gender-specific sexual contact coefficients during pre-couple and extra-couple sexual activity (*c*).

We refer to $c$ as a sexual contact coefficient rather than a contact rate because it incorporates both the rate of contact with casual partners and also those partners’ probability of being infectious relative to the average population (where average population infectious prevalence is represented by *P*(*t*)). Although we do not model sexual network structure explicitly, our estimated sexual contact coefficients reflect risk associated with network structure: any increased risk from non-stable partners due to network characteristics (e.g., partner acquisition rates, concurrency, age-disparate relationships, other sexual network characteristics) would lead to greater rates of pre-couple or extra-couple transmission and, thus be reflected by greater fitted contact coefficients. Section 3a of the Supplementary Information provides a more in-depth discussion of the assumptions underlying this conceptualization of the transmission process.

Further model assumptions include that infected individuals die of AIDS according to empirically derived survival distributions^40^. This accounts for an important survival bias—couples observed during a survey are only those in which both partners survive up to the survey date^38^. We also assumed that newly infected individuals are five times as infectious for a two month-long acute phase post-infection^41^. We assumed that all individuals were infected at the same gender-route specific rates and with the same sexual contact coefficients (homogeneity assumption) due to computational obstacles to fitting a heterogeneous model. However, in a sensitivity analysis, we assessed the robustness of our results to this assumption by simulating models that included individual-level heterogeneity and between-partner assortativity therein.

### Estimating Transmission Rates and Sexual Contact Coefficients by Fitting the Model to Data

To formally estimate these six parameters (two transmission rates and four sexual contact coefficients) we fit the above model to the DHS data sets. To do so requires calculating the likelihood of the observed data for given parameters. We did this by iteratively calculating the probability of each partner’s serostatus and survival for each month of sexual activity from their sexual debuts until their DHS interview date. This yields a likelihood for each couple–i.e. given a couple’s relationship history and values for the six parameters, the probability that the couple exhibited the serostatus that they actually exhibited in the DHS. We used Bayesian Markov Chain Monte Carlo to fit this model to the data from each country, yielding the transmission rates and sexual contact coefficients that maximized the joint probability of all observed couples being alive and exhibiting their observed serostatus on their DHS survey date. The ten West African countries analyzed exhibited very few HIV-positive individuals in their sampled populations. This made it difficult to fit our model. We thus pooled these countries together for analysis.

Our method does not estimate when (seroconversion times) or how (transmission route) each infected individual was infected. Rather, fitted transmission rates and contact coefficients reflect the estimates that are most consistent with observed data while integrating uncertainty in all infected individuals’ seroconversion times and routes of infection. All results shown are medians of posterior distributions with 95% credible intervals. We assessed the accuracy and precision with which we could estimate these six parameters by fitting this model to data simulated in a separately coded model in which the true underlying parameters were known.

**Figure 2:**
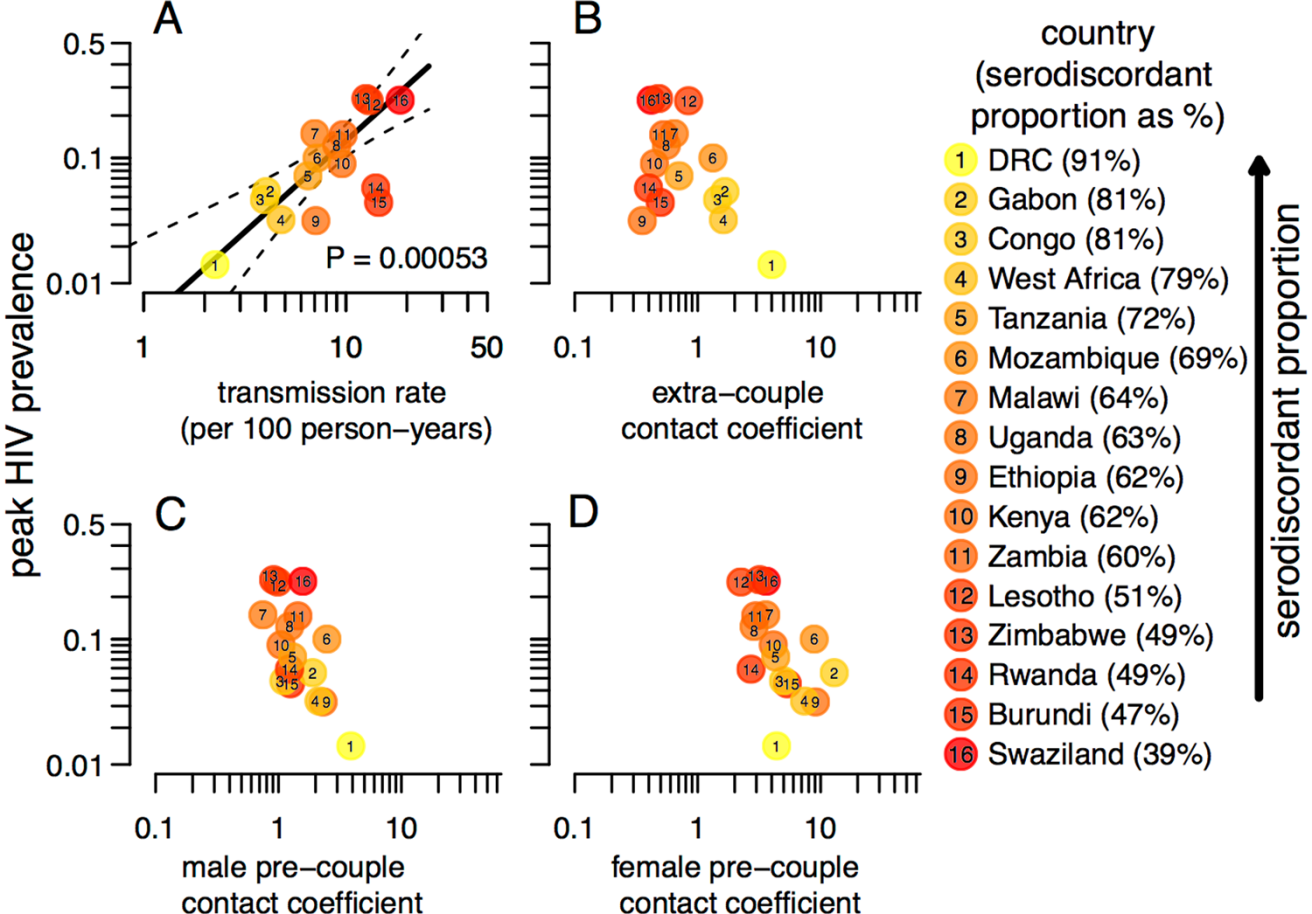
HIV peak prevalence versus the geometric mean (taken due to similarity) of male and female (**A**) HIV transmission rates and (**B**) extra-couple contact coefficients, and versus (**C**) male and (**D**) female pre-couple contact coefficients. Colors indicate SDP. Solid and dashed lines show the weighted linear regression line and 95% confidence intervals, respectively, for the best model as chosen via AICc (models with *Δ*AICc > 2 compared to the best model are rejected). Univariate models (not shown) corresponding to panels (**B**–**D**) yielded *Δ*AICc = 9.39, 5.19, and 4.27, respectively, while the full multivariate model including all predictors yielded *Δ*AICc = 5.8 (see Table 3 for further detail on each model’s results). The range of SDPs shown here reflects only those couples included in the model analysis (i.e. excluding those with missing relationship data) and thus differs slightly from that given in the main text.

In our previous work^38^ (in particular, Fig. 2 and the Discussion), we gave intuition for why estimation of these six parameters is possible through the model fitting procedure described above. Here, we briefly provide two complementary ways to understand why these parameters are estimable from these data. For the first way, consider how each parameter affects couple serostatus patterns. For instance, if male-to-female transmission rates are higher, we should see fewer male-positive serodiscordant partnerships, especially amongst couples that have been together for a long time. Similarly, if men have high pre-couple contact rates (lots of risky casual sex before entering into a stable couple), this would increase the proportion of recently formed couples that are male-positive serodiscordant, particularly amongst men who had long periods of sexual activity prior to couple formation. For the second way, consider how two partners’ relationship history can inform when an infected partner is likely to have been infected. Importantly, the data we analyze are before ART was available in these countries or when ART coverage was extremely low (e.g. Fig. 1 in^38^). Thus, any infected individuals sampled in these DHS surveys were likely to have been infected in the previous decade; otherwise they would have died before the survey. So for serodiscordant couples that formed 15 years ago, the infected partner was extremely likely to have been infected extra-couply, not pre-couply (because they would likely already be dead if infected that long ago) or within-couply (because their partner is uninfected).

### Meta-Analysis of Drivers of Variation in HIV Prevalence

Our estimated transmission and sexual contact parameters varied significantly among countries (see Results). This implies that some parameters must vary between countries in ways that explain their distinctive couple serostatus patterns and prevalences. To explicitly assess which parameters drive differences in prevalence, we performed a meta-analysis of the estimated parameters from each of our fitted country-specific transmission models. We regressed peak HIV prevalence (estimated by UNAIDS^1^) against our estimates of transmission and sexual contact parameters in weighted univariate regressions and in a weighted multivariate regression including all predictors. We also conducted a sensitivity analysis in which the dependent variable was HIV prevalence from the analyzed DHS data instead of UNAIDS-estimated peak prevalence.

Because it is a proportion, prevalence was modeled on log-odds scale; the explanatory variables (the six parameters) are all rates, and were consequently modeled on a log scale. Because estimated male and female transmission rates and extra-couple contact coefficients were similar within each country, we used the geometric means of male and female estimates for each country as explanatory variables to reduce collinearity. Pre-couple contact coefficients differed more between genders, so we included them as separate predictors. We then selected the best from among these models using Akaike’s Information Criterion corrected for small sample sizes (AICc)^42^. Since the predictors are estimated parameters each with their own uncertainty, we weighted each country in the regressions by the inverse variance of the predictor (for the multivariate model, by the geometric average variance across predictors).

### Counterfactual Simulations of Couple Serostatus Dynamics

To better understand the estimates resulting from our model fitting procedure and their regression against prevalence, we conducted simulations to elucidate how each parameter affects couple serostatus dynamics. In these simulations, we generated 100,000 couple relationship histories (i.e., sexual debuts, couple formation dates, and ages) simulated to represent the relationship histories actually observed for each country’s DHS (Figure S1). Using the transmission parameters estimated for each country above, we simulated transmission and mortality events starting from each couples’ sexual debuts up until their interview date. This allowed us to create trajectories of couple serostatus states for each couple over calendar time (Figure S2). We summarized the evolution of couple serostatus patterns over the course of the epidemic by plotting SDP over calendar time.

We then conducted counterfactual simulations, in which we varied one parameter at a time and simulated transmission and mortality for those same relationship histories, plotting the effect on the SDP trajectory for each country. Specifically, holding all other parameters constant, we varied male and female transmission rates, and pre- and extra-couple contact coefficients from zero to ten times their estimated values for each country.

We also used these counterfactual simulations to explore the effects of between-individual heterogeneity in infection risk, and of between-partner assortativity in risk of infection. We modeled heterogeneity as each individual having a lognormally distributed risk factor that elevated or reduced their risk of transmission through any route. We modeled assortativity as between-partner correlation in these risk factors.

We neither considered heterogeneity nor assortativity in our fitted model because doing so is computationally challenging since it would require presuming each observed individual exhibits some unobserved risk factor that may or may not be correlated with their partner’s unobserved risk factor. In contrast, simulating (versus fitting) heterogeneity and assortativity is relatively straightforward, since we can simulate partners with higher or lower risk with or without between-partner correlation, and then explore the subsequent effects on couple serostatus dynamics. Assortativity could be induced by host genetic factors that vary between sub-populations, by partners exhibiting similar co-infections that affect HIV infectivity or susceptibility, or simply by variation in prevalence between subpopulations such that individuals in geographic proximity, or similar social circles, both exhibit more similar infection risk and are more likely to form partnerships.

### Data Availability

The *raw* data that support the findings of this study are available from the Demographic and Health Surveys (www.measuredhs.com) upon request. The *cleaned* datasets containing only observations and variables analyzed during the current study are available at https://github.com/sbellan61/SDPSimulations along with all analyses performed in this manuscript.

## Results

### Estimated transmission rates and sexual contact coefficients

Table 1 displays summary statistics of the couples data analyzed here, as well as two additional variables indicative of risk behavior (condom use at last intercourse, number of lifetime sexual partners). Table 2 reports our estimated transmission rates, which span the range of estimates reported by serodiscordant couple cohort studies in low-income settings^39^ and vary substantially across countries. Between-country variation in these estimated transmission rates may reflect variation in the prevalence of risk factors that affect transmission. These include male circumcision, interacting co-infections, host and viral genetics, and likely many other known or unknown factors that affect HIV infectivity or susceptibility. We also found substantial differences between genders within a few countries. Variation in the ratio of transmission to males versus to females and its inconsistency across regions has been observed in previous studies^16^ and could be due to, for instance, variation in circumcision prevalence between sites (which reduces male but not female susceptibility). Our estimates of the pre- and extra-couple contact coefficients for each country and gender (Table S3) also varied widely, which likely reflects differences in sexual network characteristics between countries.

**Table 1:** Summary of Demographic Health Survey couples data analyzed with averages shown by country.

Country	age		age difference (M-F)	age at sexual debut		duration of sex before couple formation		partnership duration	condom used at last sex (%)		# lifetime sexual partners	
	M	F	—	M	F	M	F	—	M	F	M	F
Burundi	36.2	31.3	4.9	22.2	19.4	3	0.9	11	2	2.1	2.0	1.1
Congo	35.8	30.1	5.7	16.1	15.4	10.2	5.1	9.6	13.6	8.6	14.8	3.8
DRC	36.8	30.4	6.4	18.2	16.3	7.3	2.8	11.3	3.2	2.2	9.6	2.1
Ethiopia	36.2	29.5	6.7	20.9	16.6	3.4	1	11.9	1.3	0.9	2.8	1.1
Gabon	38.4	32.3	6.2	16.7	15.6	11.3	6.3	10.4	18.5	14.7	21	4.5
Kenya	37	31	5.9	17.5	17.3	8.3	2.6	11.2	4.6	2.8	7.9	2.0
Lesotho	36.3	30.8	5.5	19	17.7	5.8	1.7	11.4	14.8	16.6	9.6	2.2
Malawi	34.3	29.2	5.1	18.2	16.6	5.1	1.7	11	9.4	5	3.6	1.5
Mozambique	34.8	29.2	5.6	17.9	16.3	6.3	2.3	10.5	8.9	6	5.1	1.9
Rwanda	36.2	32.2	4	21	20.1	4.3	1.3	10.8	4.3	3.7	2.7	1.2
Swaziland	36.6	30.6	6	19.8	17.3	8.2	4.7	8.6	23.8	24.3	8	2.1
Tanzania	34.9	29.3	5.6	19.1	17.2	5.9	2.2	10	7.1	5	6.1	1.9
Uganda	35.9	30.1	5.8	18	16.3	6.8	2.6	11.1	4.6	4.3	7.6	2.2
WA	37.7	29.6	8.1	19.9	16.3	6.8	2.3	11.1	7.5	3.3	7.2	2.0
Zambia	35.7	30	5.7	17.6	16.5	6.9	2.3	11.1	13.7	7.7	6.5	1.8
Zimbabwe	35.4	29.8	5.6	20	17.8	5	1.6	10.4	8.7	4.6	5.8	1.6

All durations are given in years.

**Table 2:** Estimated HIV transmission rates per 100 person years (95% credible intervals) obtained by fitting a model of transmission into and within couples to couples serostatus and relationship history data from Demographic and Health Surveys.

	Country	*β*	Country	*β*
male	Burundi	13 (6.1, 25)	Mozambique	9.6 (5.7, 14)
female		16 (8.1, 30)		5.5 (3, 9.1)
male	Congo	4.3 (2, 8)	Rwanda	15 (8.3, 23)
female		3.6 (1.5, 7.2)		14 (9.7, 19)
male	DRC	2.4 (0.41, 7.9)	Swaziland	20 (11, 32)
female		2.1 (0.36, 7)		17 (9.6, 29)
male	Ethiopia	6.8 (3.7, 11)	Tanzania	9.2 (6, 13)
female		7.4 (4.4, 11)		4.7 (2.6, 7.4)
male	Gabon	4.7 (2.2, 8.4)	Uganda	11 (7.1, 16)
female		3.5 (1.4, 7.6)		7.5 (4.5, 11)
male	Kenya	9.8 (5.6, 15)	West Africa	4.5 (2.5, 7.3)
female		9.5 (5.1, 16)		5.2 (3.1, 7.4)
male	Lesotho	13 (6.4, 21)	Zambia	12 (6.8, 18)
female		14 (8.6, 19)		8 (4.7, 12)
male	Malawi	8.3 (4.7, 13)	Zimbabwe	15 (9.8, 20)
female		6.1 (3.6, 8.9)		11 (7.7, 15)

Gender labels indicate transmission rates *to* individuals of that gender.

### Drivers of between-country variation in prevalence

In our meta-analysis of peak HIV prevalence against these estimated parameters, we determined that the best model (according to AICc selection; Table S4) was a univariate model in which the transmission rate was the only explanatory variable (Fig. 2). We found that the transmission rate explained the majority of the between-country variation in peak HIV prevalence (R^2^ = 59%) and remained significant in the full multivariate model (Table 3). In contrast, all models containing pre-couple and extra-couple contact coefficients were far inferior and these explanatory variables were not statistically significant predictors of peak HIV prevalence in the multivariate model. This means that countries with higher estimated extra-couple or pre-couple contact coefficients did not necessarily have higher HIV prevalence. In contrast, countries in which we estimated higher transmission rates did consistently exhibit higher prevalence. These findings were similar in a sensitivity analysis with DHS prevalence (averaged for each country) used as the dependent variable instead of UNAIDS-estimated peak prevalence (Figure S3).

**Table 3:** Regression results.

	Univariate (95% CI)	P	Multivariate (95% CI)	P
transmission rate	**1.5** (**0.79, 2.2)**	**0.00053**	1.7 (0.5, 2.9)	0.0096
extra-couple contact coefficient	−0.86 (−1.7, −0.026)	0.044	0.64 (−0.29, 1.6)	0.16
male pre-couple contact coefficient	−1.5 (−2.5, −0.52)	0.0058	−1.1 (−2.4, 0.15)	0.077
female pre-couple contact coefficient	−1.2 (−2, −0.47)	0.0038	0.11 (−1.1, 1.3)	0.84

Effect sizes and 95% confidence intervals from univariate and multivariate regressions of (log-odds) peak HIV prevalence versus the HIV transmission rate $\beta$, extra-couple contact coefficient (${c}_{e}$), and male and female pre-couple contact coefficients (${c}_{M,p},{c}_{F,p}$) as fitted from a mechanistic model assuming homogeneity in infectiousness and susceptibility within countries. All explanatory variables were regressed on a log scale. The best model (bold) as chosen via AICc model selection (Table S4) was the univariate model containing only the transmission rate ($\Delta$ AICc = 4.27 above second best model).

We examined whether this finding might be modulated by how infectious we assumed the acute phase to be relative to the chronic phase. Our results were quite robust to the assumed acute phase relative infectivity, with greater values leading to lower estimated chronic phase transmission rates (consistently across countries such that between-country patterns were not affected) but negligibly affecting estimates of contact coefficients (Figure S4, Table S5).

### Counterfactual simulations to understand the drivers of couple serostatus dynamics

**Figure 3:**
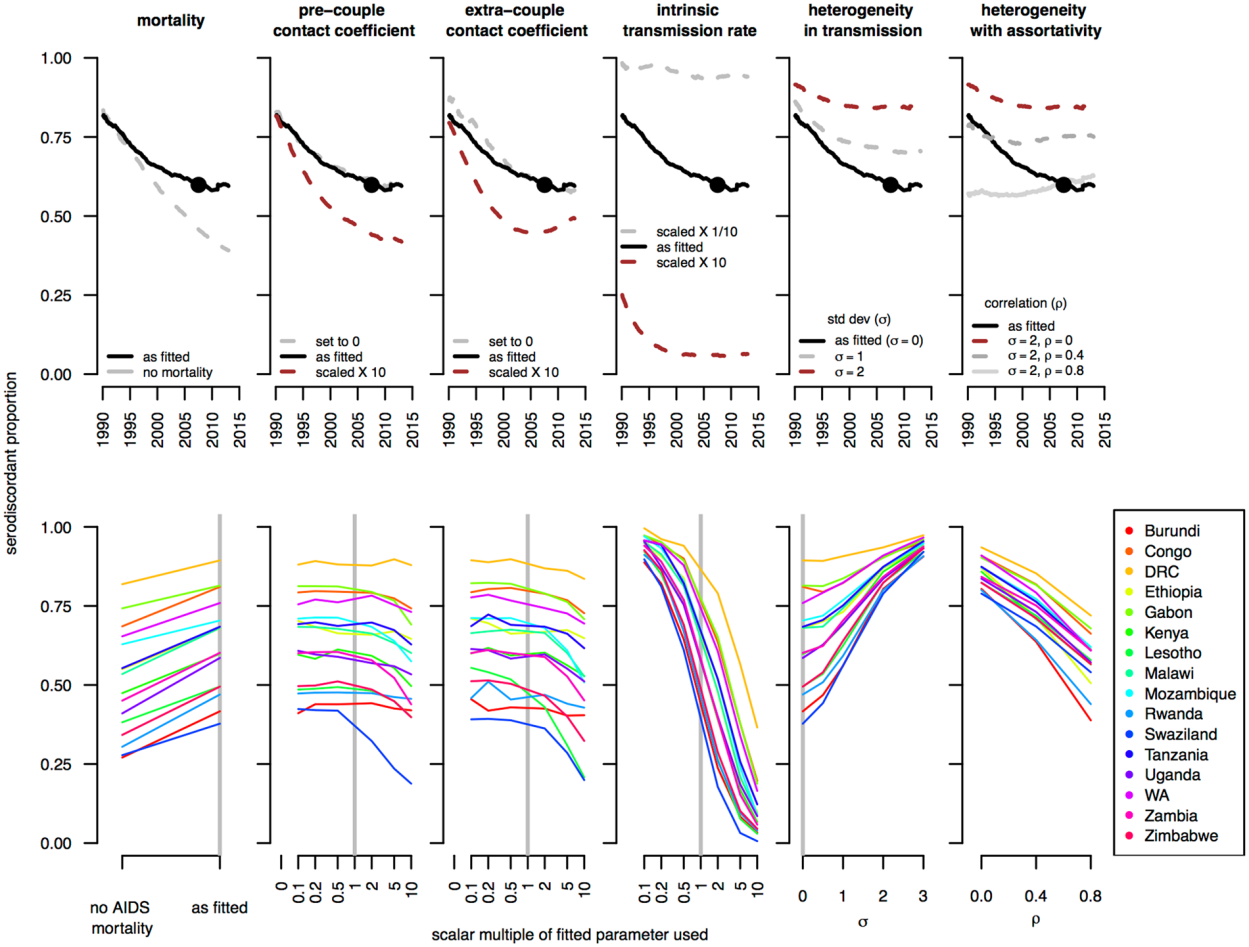
The effects of AIDS mortality and HIV transmission routes on the serodiscordant proportion (SDP). In the top row, the thick black lines show estimated historical SDP as fitted to the 2007 Demographic Health Survey in Zambia (chosen as a representative example); the black points indicate the SDP observed in the survey; gray lines show simulated SDP under various counterfactual scenarios: without AIDS mortality, with scaled pre-couple or extra-couple contact coefficients, with scaled transmission rates, or with increasing amounts of heterogeneity ($\sigma$ indicates standard deviation of lognormally distributed risk deviate) or between-partner assortativity ($\rho$ indicates inter-partner correlation in risk deviate) in infection risk. The lower row shows for each country the sensitivity of the simulated SDP (Y axis) in 2008 to variation in these same factors (on X axis). Colors indicate each country analyzed. Vertical gray bars highlight simulations parameterized as fit to the individual-level data on couple serostatus and relationship history. The serodiscordant proportion is extremely sensitive to the transmission rate, heterogeneity or assortativity in infection risk; slightly affected by AIDS mortality; and relatively insensitive to pre-couple and extra-couple contact coefficients. All simulated SDPs represent simulations of 100,000 couples.

Our counterfactual simulations further clarified the above results, by showing that SDP is substantially affected by some parameters while negligibly affected by others. Some parameters, if varying between countries would in theory cause between-country variation in prevalence; but because they minimally affect SDP, they would not induce the covariation between SDP and prevalence that is observed (Fig. 2) and thus are not viable explanations for the observed prevalence variation (Figure S5). Specifically and as shown in Fig. 3, SDP was elevated by AIDS mortality, slower transmission rates, higher inter-individual heterogeneity in risk to infection, or less between-partner assortativity in risk of infection. SDP was minimally affected by pre-couple or extra-couple sexual contact coefficients.

To understand the results in Fig. 3, consider that the factors that affect SDP are those that modify the duration that a couple spends serodiscordant or concordant positive. Slow transmission rates cause couples to spend long periods serodiscordant. Heterogeneity in infection risk leads to the accumulation of low-risk couples that stay serodiscordant for very long times, and increases SDP. Assortativity in infection risk, in which higher infection-risk individuals are more likely to partner with each other, decreases SDP because it increases the rate at which couples form already concordant positive (skipping the serodiscordant state entirely) or the rate at which couples transition from serodiscordant to concordant positive (since infected individuals are likely to have partners highly susceptible to infection). Death of one partner also causes the cessation of a couple serostate. By the time a partner is dying of AIDS (approximately a decade post-infection), it is likely they have exposed their partner for a long time. This means that SDP will be lower in couples with than those without an AIDS-stage partner. Thus, AIDS mortality disproportionately removes concordant positive couples and, consequently, increases SDP. Finally, the reason sexual contact coefficients minimally affect SDP is because they play a relatively small role in determining how long a couple stays serodiscordant. This is because within-couple transmission is the dominant means through which a serodiscordant couple becomes concordant positive; and so change in the contact coefficients only minimally change the duration of the serodiscordancy state.

## Discussion

For decades, the HIV community has struggled to explain the variation in epidemic prevalence across SSA. Efforts to disentangle the epidemiological significance of biological and behavioral factors have been criticized as too reliant on mathematical transmission models^43^. These models—built from published transmission rates from a few well-documented cohorts and hypothetical sexual network configurations^5,15,44,45^—have demonstrated that both individual-level risk factors and sexual network characteristics have the *potential* to affect epidemic severity. However, the divergent methods and populations used to estimate model parameters and the scarcity of empirically derived sexual networks have precluded a systematic partitioning of the relative influence of each factor on epidemic prevalence. Other, cross-sectional analyses of empirical data have found correlations between prevalence and biological or self-reported behavioral risk factors^19,46–48^; but such studies are unable to establish causality and are susceptible to social desirability bias. Chemaitelly *et al*.^8^ estimated transmission rates in several countries with DHS data similar to that considered here by fitting a compartmental transmission model^8^; but their results’ reliance on fitting to population-level metrics instead of individual-level data allows for a highly susceptibility to confounding.

We suggest that the ideal approach to address this question would interpret data using mechanistic models capable of distinguishing distinct causal pathways from each other by identifying nonlinear patterns in data that can be causally explained by some pathways and not others. To that end, we fit a mechanistic couples transmission model to representative samples from 15 SSA countries (and one region) and simultaneously estimated multiple components of transmission. Due to computational challenges, we were only able to fit homogenous models that do not account for individual heterogeneity or assortativity in infection risk. We found that when interpreted through the lens of these homogeneous models, couple serostatus patterns strongly suggest that the principal driver of between-country variation in both HIV prevalence and SDP is between-country variation in the transmission rate (Fig. 2 and Table 3), and not between-country variation in sexual contact patterns as previously suggested by other studies^29^.

In a sensitivity analysis, however, we show that this conclusion may not be robust to heterogeneity in infection risk or between-partner assortativity therein. These processes also affect couple serostatus patterns, and it is possible that they could vary between countries in ways that would weaken the above results. Future work that can estimate between-country differences in heterogeneity and assortativity will be necessary to more robustly determine which mechanisms can plausibly explain between-country variation in couple serostatus patterns and epidemic prevalence. It is important to keep in mind that such methods may reveal that it is not possible to distinguish these hypotheses with the available survey data.

In an earlier study fitting the same models to the DHS, we previously demonstrated that risky network characteristics (such as concurrent partnerships, partner acquisition rates, age-disparate relationships) drive the majority of HIV incidence *within* all countries analyzed^38^. This may be true for most or all sexually transmitted infections, as sexual contacts with numerous non-stable partners fuels transmission far more than the same number of contacts between stable partners^49^. Nevertheless, we point out a nuanced distinction; even if risky sexual network characteristics are responsible for the majority of transmission within each country, it still is plausible that differences *between* countries are not explained by differences in network characteristics, but instead by differences in underlying transmission rates. Future work examining between-country differences in epidemic severity should pay careful attention to this distinction.

While our analysis and results exhibits some similarity to that of Chemaitelly *et al*.^29^, we have also made several advances. First and foremost, here and previously^38^ we leverage individual-level relationship history and serostatus data to understand couple serostatus dynamics, rather than relying on population-level statistics. Without individual-level data on relationship duration, an analysis cannot accurately represent the denominator of time at risk when estimating transmission between serodiscordant partners. We believe this explains why our transmission rate estimates (ranging 2.1–20 per 100 person-years) were about half of those of Chemaitelly *et al*. (ranging 4.4–41 per 100 person-years^8^) for each country, though the country-order of transmission rates compares closely between the two analyses (Table S6, Figure S6). The other main advances of our analysis comprise the explicit estimation of pre-couple and extra-couple contact rates for each country; the systematic assessment of which of several factors best explain between-country variation in prevalence; and examination of how individual heterogeneity in infection risk and between-partner assortativity in infection risk alter serostatus dynamics.

If in fact regional variation in transmission rates drives a large proportion of the observed variation in HIV prevalence, this has important implications for the prospects of various interventions. For instance, this would suggest that the level of effort allocated to known effective interventions (e.g., ART coverage, medical male circumcision coverage) necessary to reduce incidence below a given threshold could differ dramatically between two communities, even if they exhibit similar sexual network dynamics.

### Model assumptions and limitations

Our model was designed to capture observed couple serostatus patterns, given limitations of the available data. We made many simplifying assumptions and omissions. In addition to the main sensitivity analyses highlighted above, we additionally assess the robustness of our conclusions to other simplifications below.

While we estimated country-specific transmission rates and sexual contact coefficients, we did not explicitly estimate the drivers underlying the observed between-country variation in these parameters. Such drivers could include between-country variation in male circumcision, interacting co-infections, or host and viral genetics. Data at the individual level were not available on these factors from the DHS (of these factors, male circumcision status was available for some but not all of the DHS analyzed) and so we were unable to include them. Importantly, it is highly likely that not all risk factors for HIV susceptibility or infectivity have been characterized. Thus, there may be many unknown factors that explain geographic variation in transmission rates. Furthermore, even if we had individual data on some of the known risk factors, it remains poorly understood how the myriad factors known to affect HIV transmission interact with each other (i.e. are relative risks simply multiplicative or are there interactions between known risk factors that further amplify or mitigate risk). Thus, rather than try to tease apart the all the constituent factors affecting transmission, we aim only to characterize the average transmission rate at the country level.

Most HIV modeling studies in Africa have relied on a single transmission rate for the continent, estimated from one or more serodiscordant couple cohort studies. We estimate transmission rates and contact coefficients at the country-level to provide greater spatial resolution into HIV transmission rates than previously available. Nonetheless, recent work has highlighted the substantial *subnational* variation in HIV prevalence^18,50^, suggesting that biological and behavioral factors vary greatly at even smaller scales. We did not aim to estimate subnational variation in transmission rates both due to computational challenges and due to the smaller sample sizes available within the DHS when considering subnational levels. However, we do believe that subnational studies of variation in transmission rates are warranted in future work.

Higher dissolution rates in HIV-status aware serodiscordant couples^51^ (compared to other couples) could increase SDP by dissolving serodiscordant couples before they become concordant positive. Given the scarcity of data, we were unable to assess whether this might explain between-country variation in SDP, though a preliminary analysis suggests it is unlikely (Supplementary Text Section A7, Figure S7). Indeed, we believe it is unlikely that such dissolution would explain significant variation, given the relatively low levels of partner serostatus awareness during the period of study^52^ and lack of evidence that awareness differed substantially between countries during the period considered.

Our model includes the treatment as prevention effect of ART on reducing transmission rates by modeling the risk of infection from casual partners as a function of the proportion of the population that is HIV-infected and not on ART. But, because treatment status was not ascertained in these DHS surveys, we could not model the effects of ART on within-couple transmission or on survival times of infected partners. In a previous sensitivity analysis^38^, we showed our results are nonetheless robust, since, for the period of study and until recent policy changes^53^, most treated individuals would have become eligible for ART only after a long period of exposing their partner to infection. This holds true even considering prevention of mother-to-child transmission (PMTCT) programs, which expanded ART coverage for pregnant and postpartum women. WHO guidelines pre-2010 offered ART to pregnant women starting 28 weeks into pregnancy and lasting up until 7 days postpartum. WHO’s 2010 guidelines added option B, in which women are given the opportunity to stay on ART until the end of breastfeeding. Thus, for the period considered PMTCT would have only led to ephemeral reductions in transmission from women to their male partners, and would have had a limited impact on survival.

With recent increases in ART coverage, however, we expect the relationship between SDP and HIV transmission rates to have become increasingly complex because—depending on who receives ART and when they receive it in their course of disease—ART can either act to reduce SDP (by increasing the life expectancy of concordant positive partners^54^) or to increase SDP (by reducing infectiousness^55^ and thus increasing the time couples spend serodiscordant).

For a subset of the countries analyzed, the DHS conducted surveys at multiple years within the period of analysis. For each of these countries, we chose to pool all DHS surveys across years for analysis. We made this choice for two reasons. First, SDP was relatively stable over time across surveys (Figure S8). Second, we did not believe we would have sufficient sample size to pick up evidence of temporal variation in transmission rates or contact coefficients, particularly since cross-sectional couple serostatus patterns observed in a survey reflect transmission dynamics occurring over the preceding decade. In estimating a single set of parameters for each country, we implicitly assumed that HIV transmission rates and sexual contact coefficients were constant over time. Declining HIV prevalence in several countries, however, has been attributed to behavioral changes in response to interventions or overall HIV awareness^56^. Decreasing pre- or extra-couple transmission in recent years, while holding transmission rates constant, could temporarily decrease the SDP (by reducing the creation of new serodiscordant couples from concordant negative couples, while pre-existing serodiscordant couples continue to become positive concordant)^30^. A decreasing transmission rate over time would cause a more direct and permanent increase in SDP. Neither of these patterns, however, appears likely to explain the tight empirical relationship between SDP and peak prevalence. On the other hand, this pattern is parsimoniously explained by between-country variation in transmission rates.

Finally, in view of the range of the DHS and the scope of our study, we necessarily excluded many couples because of missing or inconsistent data. A notable exclusion was that of all polygamous couples (given the couple-centric nature of our model), which represented a third of all couples in West Africa. Nonetheless, the low prevalence, low transmission rates and high serodiscordant proportion observed in the included (non-polygamous) couples from West African are consistent with our main findings. Previous sensitivity analyses also suggest that our results are robust to the exclusion of couples with missing data^38^ and are roughly generalizable to the couples population sampled by DHS. Furthermore, these population samples are likely to be more representative of the general population than are the intervention or cohort studies most frequently used to understand HIV transmission rates, given their narrow selection criteria and the effects of intensive study on participant behavior.

## Conclusion

Couple serostatus patterns in combination with relationship history patterns can provide insight into between-country variation in HIV epidemic prevalence across sub-Saharan Africa. That greater HIV transmission rates underlie more severe epidemics is a parsimonious explanation for the inverse correlation between the serodiscordant proportion and peak epidemic prevalence. However, heterogeneity in infection risk, and between-partner assortativity therein, may also partially explain this pattern and future work is warranted to distinguish between these hypotheses.

## Electronic supplementary material

S1
