# Supplementary material for: Couple serostatus patterns in sub-Saharan Africa illuminate the relative roles of transmission rates and sexual network characteristics in HIV epidemiology
Source: Sci Rep. 2018 Apr 27;8:6675. doi: 10.1038/s41598-018-24249-7 (PMC5923291; doi:10.1038/s41598-018-24249-7)
Supplement: Supplementary file 1 — S1 [file 41598_2018_24249_MOESM1_ESM.docx]

**Couple serostatus patterns in sub-Saharan Africa illuminate the relative roles of transmission rates and sexual network characteristics in HIV epidemiology**

Steve E. Bellan, David Champredon, Jonathan Dushoff, Lauren Ancel Meyers

**Supplementary Online Web Appendix**

**All code necessary to run this analysis is available on the GitHub repository** [**https://github.com/sbellan61/SDPSimulations**](https://github.com/sbellan61/SDPSimulations)

**Table of Contents**

**A. Detailed Explanation of Methods.**

1. **Introduction**
2. **Data Sets**
3. **Fitting Transmission Models to DHS Data (Addendum to Previous Work)**
4. **Sensitivity Analyses**
5. **Simulation Model**
6. **Counterfactual Simulations**
7. **Couple Dissolution Analysis**
8. **Drivers of Between-country Variation in Prevalence**
9. **Reproduction of Results and Extension to the Model**
10. **Online Supplementary Files**

**B. Cited References**

**C. Supplemental Tables**

1. **Summary of Demographic & Health Surveys data sets and grouping for analysis**.
2. **Summary of Demographic & Health Surveys data analyzed and inclusion criteria.**
3. **Estimated HIV transmission rates.**
4. **AICc Model Selection Table**

**D. Supplemental Figures**

1. **Simulated relationship histories and serodiscordant proportions.**
2. **Survival-inflated copula model of couple relationship histories for Zambia.**
3. **Serodiscordant proportion versus relationship duration and serodiscordant proportion by country.**
4. **Counter-factual versus observed transmission patterns.**
5. **Sensitivity of estimated transmission coefficients to assumed acute to chronic phase relative hazard.**
6. **Sensitivity of estimated sexual contact coefficients and intrinsic transmission rate to assumed acute to chronic phase relative hazard.**

**A. Detailed Explanation of Methods.**

**1. Introduction**

In previous work^1^ we built a model to interpret cross-sectional data collected by the Demographic & Health Survey (DHS) on human immunodeficiency virus (HIV) serostatus and other variables in stable, cohabiting couples. This model used information on couple serostatus, partnership duration, and timing of sexual debuts to estimate the probability that an HIV positive individual was infected prior to couple formation, during the duration of their partnership from their partner, or during their partnership from extra-couple intercourse. For brevity in the rest of this appendix, we collectively refer to couples married or living as married (i.e. stable, cohabiting couples) as a “couple”.

The analyses described here build from this previous work and refer to that manuscript as well as the accompanying web appendix. We therefore only describe changes made to the previous model (notably the addition of a variably infective acute phase) as well as the new simulation model built for this analysis.

This appendix serves two distinct purposes: (1) to include supplementary results and sensitivity analyses we could not fit in the main text, and (2) to provide a technical description sufficiently thorough for the interested reader to reproduce or build from the analyses. Readers that are uninterested in the technical detail are advised to skip Sections 3, 5, and 6 and the mathematical parts of Section 4.

Please note that while the data sets (except for restricted subgroups) and code have been provided as supplementary material, the analyses therein were computationally intensive and run in parallel on Lonestar5 (a high-performance computing system of the Texas Advanced Computing Center). The interested reader should plan on acquiring the necessary computational resources if planning to reproduce or extend the results.

**2. Data sets**

**2a. Demographic and Health Surveys**

All available DHS data sets in sub-Saharan Africa that tested stable, cohabiting couples for HIV were downloaded and cleaned. These included data sets already merged into couple line lists by DHS, as well as individual line list data sets that we merged into couple line lists based on partner identifiers. We are happy to share both the cleaned data sets as well as the computer scripts used to clean them with any interested readers. The following list describes the inclusion criteria for our analysis as well as data imputation methods used for inconsistent data:

- We excluded couples for which HIV serostatus was not available for one or both partners.
- Our model relies heavily on knowledge of a couple’s relationship duration. DHS surveys, up to DHS phase V (i.e., all surveys before 2009), however, do not ask couples how long they have been together. Interviewers do ask if interviewees are in their first couple, and when they entered their first couple. Thus, we restrict our analysis to couples in which at least one individual is in the first stable, cohabiting couple of their lifetime because we can infer relationship duration from the latter question.
- We removed all polygamous couples from the analysis because such couples require consideration of more complex within-partnership transmission dynamics.
- When the male and female’s listed date of couple formation differed we used the mean. We excluded couples from the analysis if this discrepancy was greater than 25% of the mean or if either partner reported a date of couple formation that occurred before they were 8 years old.
- We excluded couples that included individuals giving an age of sexual debut (i.e. age at first intercourse) as more than a year after the date of couple formation.
- For individuals who listed the age at sexual debut as within 1 year after the date of couple formation, we assumed that they began having intercourse when they formed the stable, cohabiting couple.
- Couples with individuals stating that their sexual debut occurred at less than 5 years old were also excluded from the analysis.

We did not include data on condom usage or other behavioral risk factors reported in the DHS because all questions only asked about behavior within the last 12 months. We believe that such data are inappropriate to use as indices of risk for relationship histories that are several years or decades long. We assessed the sensitivity of our results to the exclusion of couples due to missing HIV serostatus as outlined in our model section below. Tables S1-2 summarizes the surveys used and the data cleaning process.

**2b. UNAIDS Data**

See section **2b** in the previously published online web appendix. Our results have been updated using the UNAIDS models cited in the 2013 Global Report ^2^.

**2c. HIV Survival Time Data**

See section **2c** in the previously published online web appendix.

**3. Model Structure**

**3a. Transmission routes and the hazard of infection**

**Due to the similarity between the model used in this manuscript and that in Bellan et al. (2013), this section is nearly identical^[[1]](#footnote-1)^ to section 3a of the online web appendix for Bellan et al. (2013). The primary difference is the addition of an acute HIV phase after infection during which individuals are extremely infectious**^3^**. Text (but not equations) that is new in this manuscript relative to the former manuscript is printed in bold to allow the reader to identify model components that are new to this manuscript.**

For each partner in a couple, we assume there are three possible routes of infection (Figure 2 in main text, Figure S1):

1. they were infected prior to formation of the current partnership (pre-couple transmission),
2. they were infected during their partnership from extra-couple intercourse (extra-couple transmission),
3. they were infected during the partnership by their partner (within-couple transmission).

Each couple is only observed once in a cross-sectional survey. Thus, the serostatus of a couple can either be $\left\{ M^{-},F^{-} \right\},\left\{ M^{+},F^{-} \right\},\left\{ M^{-},F^{+} \right\},\left\{ M^{+},F^{+} \right\}$. The probability that an infection occurs via any of the three routes of transmission is a function of the hazard of infection through that route, $\lambda$, defined to be the rate of new infections per susceptible individual per month. Susceptible individuals are infected when they have intercourse with another individual, that individual is infectious, and during intercourse HIV is successfully transmitted to them.

**We then allow for six different hazards reflecting all gender-route combinations. The six transmission coefficients are therefore before (b) couple formation (**$\boldsymbol{\lambda}_{\mathbf{M,b}} \mathbf{and}\boldsymbol{\lambda}_{\mathbf{F,b}}$**); during the partnership (p) by a positive partner (**$\boldsymbol{\lambda}_{\mathbf{M,p}} \mathbf{and}\boldsymbol{\lambda}_{\mathbf{F,p}}$**); or during the partnership by engaging in extra-couple (e) intercourse (**$\boldsymbol{\lambda}_{\mathbf{M,e}} \mathbf{and}\boldsymbol{\lambda}_{\mathbf{F,e}}$**).** Note that all subscripted gender indices always refer to the partner who is at risk of transmission so that $\lambda_{M,p}$ is the transmission coefficient for transmission to a male from his female partner.

**While the previous manuscript relied on a model parameterization based on transmission coefficients (i.e.,** $\boldsymbol{\beta}_{\mathbf{M,b}}\boldsymbol{,}\boldsymbol{\beta}_{\mathbf{F,b}}$**,** $\boldsymbol{\beta}_{\mathbf{M,e}}\boldsymbol{,} \boldsymbol{\beta}_{\mathbf{F,e}}$**,** $\boldsymbol{\beta}_{\mathbf{M,p}} \mathbf{and}\boldsymbol{\beta}_{\mathbf{F,p}}$**), the current manuscript uses a parameterization that allows us to disentangle the effect of sexual network characteristics on the force of infection from other behavioral and biological factors. The two parameterizations yield the same model, however, and all parameters can be simultaneously estimated by tracing them during Markov Chain Monte Carlo sampling.**

**Specifically, each hazard can be broken down into** $\boldsymbol{\lambda}\mathbf{=}\boldsymbol{c}\boldsymbol{\times\beta}^{\boldsymbol{*}}\boldsymbol{\times P}$**, where** $\boldsymbol{c}$ **is the sexual contact coefficient,** $\boldsymbol{P}$ **is the proportion of coital acts that are with an infectious partner, and** $\boldsymbol{\beta}^{\boldsymbol{*}}$ **is the hazard of transmission per unit time given an individual is engaging in a sexual relationship with an infected (stable or non-stable) partner (in the main text we refer to** $\boldsymbol{\beta}^{\boldsymbol{*}}$ **as** $\boldsymbol{\beta}$ **since we do not discuss transmission coefficients explicitly therein).** **We then define** $\boldsymbol{\beta=c\times}\boldsymbol{\beta}^{\boldsymbol{*}}$ **to be the transmission coefficient and note that our usage of ‘**$\boldsymbol{\beta}$**’ here is similar to its use in traditional SIR models** ^4^**. For within-couple transmission between stable partners, the sexual network is simply a stable pair and the hazard therefore simply equals the intrinsic transmission rate:**

$$\lambda_{M,p}=\left\{ \begin{aligned} 0 if partner uninfected \\ \beta_{M}^{*} if partner infected \end{aligned} \right.$$

$$\lambda_{F,p}=\left\{ \begin{aligned} 0 if partner uninfected \\ \beta_{F}^{*} if partner infected \end{aligned} \right.$$

**In other words, we assume that the baseline transmission rate is equivalent to the rate at which transmission occurs between members of a serodiscordant couple. In the Supplementary Material we use** $\boldsymbol{\beta}_{\mathbf{M}}^{\boldsymbol{*}}$ **and** $\boldsymbol{\beta}_{\mathbf{M,p}}$ **interchangeably (and similarly for female rates). This is a common assumption; to our knowledge and with the exception of our previous manuscript** ^1^**, all existing estimates of HIV transmission rates have been made by estimating the rate at which transmission occurs between stable serodiscordant partners** ^5,6^**.**

**For non-stable partnerships (i.e., pre-couple and extra-couple interactions), we define:**

$$\beta_{M,b}=\beta_{M}^{*}\times c_{M,b}$$

$$\beta_{F,b}=\beta_{F}^{*}\times c_{F,b}$$

$$\beta_{M,e}=\beta_{M}^{*}\times c_{M,e}$$

$$\beta_{F,e}=\beta_{F}^{*}\times c_{F,e}$$

**This breakdown of the transmission coefficient into a baseline transmission rate (**$\boldsymbol{\beta}^{\boldsymbol{*}}\boldsymbol{)}$ **and a contact coefficient (**$\boldsymbol{c}$**) also has precedent in epidemiological modeling; however, there are some nuances to our parameterization worth pointing out. First, because we do not have data on the number of coital events per unit time over the entire sexually active lifetimes of the couples analyzed, we define** $\boldsymbol{\beta}^{\boldsymbol{*}}$ **to be the transmission rate per unit time between two partners (stable or non-stable) rather than per coital event. Second and consequently, we note that** $\boldsymbol{\beta}^{\boldsymbol{*}}$ **aggregates the effects of both biological factors underlying the probability of transmission per coital act (STI and other coinfections, male circumcision, viral and host genetics) as well as coital behavior (frequency of intercourse, safe sex practices, and type of intercourse—e.g. vaginal, anal, dry sex, etc…).**

**The sexual contact coefficient (**$\boldsymbol{c}$**) includes all other sexual network characteristics that determine whether an individual is currently in a sexual relationship with a non-stable partner (i.e. partner acquisition rate) and that partner’s risk level (i.e. the probability that they are infectious, acutely infectious as a consequence of concurrency, or any other factors that make them more likely to be infectious compared to the average population prevalence). Additionally, sexual contact coefficients include any difference in coital behavior or intrinsic transmission rates between stable and non-stable partnerships. For instance, if dry sex were much more common between stable versus non-stable partners (and increased the probability of transmission), then this could be reflected by smaller sexual contact coefficients.**

**We also allow for increased infectivity during the acute phase such that for the 2 months after an individual has been infected, their partner’s risk of transmission is elevated from** $\boldsymbol{\beta}_{\mathbf{M,p}}$ **to** $\boldsymbol{a\times}\boldsymbol{\beta}_{\mathbf{M,p}}$ **or** $\boldsymbol{\beta}_{\mathbf{F,p}}$ **to** $\boldsymbol{a\times}\boldsymbol{\beta}_{\mathbf{F,p}}$ **for susceptible male and female partners, respectively**. **Our base analysis assumes** $\boldsymbol{\alpha}$ **= 5 based on recent work**^7^**, but all results are examined across a sensitivity analysis of the relative hazard of acute versus chronic infectivity,** $\boldsymbol{\alpha}$**, ranging from 1-50.**

**3b. Iterating serostatus state variables through time**

We calculate the probability that individuals are infected through various routes of transmission using the rate to probability transformation $p=1-exp(-\lambda\Delta t)$ where $\Delta t$ is one month. Our general approach is to iterate the probability that a couple is in any given couple serostatus through time (month by month) while keeping track of the joint probability both individuals survive to DHS sampling. We also keep track of the cumulative probability for each route of transmission. For the $i$-th of $K$ couples we define $t_{\mathrm{msd},i}$, $t_{\mathrm{fsd},i}$, $t_{\mathrm{cf},i}$, and $t_{\mathrm{int},i}$ , *i*=1,…,*K*, to respectively be the points in time (months) of the male (msd) and female (fsd) partners’ sexual debuts, the month they formed a couple (cf), and the month during which they were sampled/interviews (int) by the DHS survey. We define $t_{\mathrm{sd},i}=\text{min}(t_{\mathrm{msd},i},t_{\mathrm{fsd},i})$ to be the time of the first sexual debut amongst partners in the $i$-th couple. Then we can iteratively calculate the probability that the $i$-th couple is in either of the four states $\left\{ s_{i},m_{i},f_{i},h_{i} \right\}$, corresponding to concordant HIV negative, serodiscordant male-HIV-positive, serodiscordant female-HIV-positive, and concordant HIV positive states.

We, however, keep track of routes of infection and so divide the latter three state variables into more specific states. We use a subscripting convention similar to the transmission coefficients above but give the route of infection for both males (first subscript) and females (second subscript), and use “-” when an individual is negative. As examples, $m_{b-,i}$ denotes a male-positive serodiscordant couple where the male was infected before partnership formation, $f_{-e,i}$ is a female-positive serodiscordant couple where the female was infected extra-couply, and $h_{pe,i}$ denotes a concordant positive couple where the female was infected extra-couply and then infected her male partner. We also keep track of the order of infection when both genders are infected—i.e. before couple formation always occurs earliest and from-partner transmission latest. When both partners are infected pre-couply or extracouply we track who was infected first using, for example, $h_{e_{2}e_{1},i}$to represent a couple in which both partners were infected before couple formation but the female was infected first. **Finally, we also keep track of acutely infected individuals as this increases the risk posed to their partners. We assume that the acute phase lasts two months**^7–9^ **and move individuals through these two month stages (i.e.,** $\boldsymbol{m}_{\mathbf{b}\boldsymbol{-,a}\boldsymbol{1,i}}$ **and** $\boldsymbol{m}_{\mathbf{b}\boldsymbol{-,a}\boldsymbol{2,i}}$ **for acutely infected male-positive serodiscordant couples in their first and second stages of the acute phase respectively). We also consequently keep track of from-partner infections that were during the acute phase (i.e.** $\boldsymbol{h}_{\mathbf{bp,a,}\boldsymbol{i}}$ **is a couple where a male was infected before couple formation and infected his partner during his acute phase). Because our model runs in discrete monthly time steps, individuals cannot infect their partners in the same month they were infected in. This means that the two month acute phase we model corresponds to the 2^nd^ and 3^rd^ months of individuals’ infections which is roughly compatible with there being several weeklong eclipse period after infection but before the acute phase during which individuals are not yet infectious** ^3^**.** This yields 27 serostatuses:

$\left\{ \begin{aligned} s_{--,i},m_{b-,a1,i},m_{b-,a2,i},m_{b-,i},m_{e-,a1,i},m_{e-,a2,i},m_{e-,i},f_{-b,a1,i},f_{-b,a2,i},f_{-b,i},f_{-e,a1,i},f_{-e,a2,i},f_{-e,i}, \\ h_{b_{1}b_{2},i},h_{b_{2}b_{1},i},h_{be,i},h_{eb,i},h_{bp,a,i},h_{bp,i},h_{pb,a,i},h_{pb,i},h_{ep,a,i},h_{ep,i},h_{pe,a,i},h_{pe,i},h_{e_{1}e_{2},i},h_{e_{2}e_{1},i} \end{aligned} \right\}$,

All individuals are assumed to be HIV negative before they become sexually active (i.e., when $T<t_{\mathrm{sd},i}$), and all serostatus states involving transmission during the couple formation have zero probabilities before the couple formation (i.e., when $T<t_{\mathrm{cf},i}$):

$$s_{--,i}\left( T<t_{\mathrm{sd},i} \right)=1$$

When ignoring the routes of transmission and only focusing on serostatuses we use the following notation,

Negative seroconcordant couples: $s_{i}=s_{--,i}$

Male positive serodiscordant couples: $m_{i}=m_{b-,a1,i}+m_{b-,a2,i}+m_{b-,i}+m_{e-,a1,i}+m_{e-,a2,i}+m_{e-,i}$

Female positive serodiscordant couples: $f_{i}=f_{-b,a1,i}+f_{-b,a2,i}+f_{-b,i}+f_{-e,a1,i}+f_{-e,a2,i}+f_{-e,i}$

Positive seroconcordant couples: $h_{i}=h_{b_{1}b_{2},i}+h_{b_{2}b_{1},i}+h_{be,i}+h_{eb,i}+h_{bp,a,i}+h_{bp,i}+h_{pb,a,i}+h_{pb,i}+h_{ep,a,i}+h_{ep,i}+h_{pe,a,i}+h_{pe,i}+h_{e_{1}e_{2},i}+h_{e_{2}e_{1},i}$

In calculating these iterative state probabilities, we also keep track of the probability that an infected couple in each of the latter three categories will be alive at the time of the DHS survey. We use a primed notation to distinguish between the probabilities that would arise if mortality were not taken into account and the probabilities that arise once the effects of these differential mortalities have been incorporated. These serostatus states, viz.

$$\left\{ s_{--,i},{m^{'}}_{b-,a1,i},{m^{'}}_{b-,a2,i},{m^{'}}_{b-,i},{m^{'}}_{e-,a1,i},\ldots\right\}$$

are thus the probabilities of the denoted serostatus and route of transmission given both partners have survived to the time of the DHS sampling event. These states have the same initial conditions as their corresponding marginal serostatus states above. Similarly, when ignoring transmission routes, we use $\left\{ s_{i},{m'}_{i},{f'}_{i},{h'}_{i} \right\}$ to denote the probability of couple serostatus and given survival to DHS sampling.

In formulating our equations, we make the assumption that the effect of non-AIDS deaths can be neglected within these AIDS classes because, unlike with AIDS deaths, we expect non-AIDS causes of death to cause couples of each serostatus to drop from the DHS sample population base at the same rate. We iterate couples through the period ${[t}_{\mathrm{sd},i},t_{\mathrm{cf},i})$ to determine the male and female probabilities, $p_{M,b}\left( T \right)$ and $p_{F,b}\left( T \right)$, respectively that each partner is infected by the beginning of their partnership. We do this for each month $T=t_{\mathrm{sd},i},\ldots,t_{\mathrm{cf},i}$. The model is iterated monthly so that monthly time steps are $\Delta t=1\mathrm{month}$. These probabilities are functions of the infectious HIV population prevalences $P_{M}\left( T \right)$ and $P_{F}\left( T \right)$ in males and females, respectively, in the country of the $i$-th couple’s residence at time $T$ (as taken from the UNAIDS models described above). Using the indicator function $I(t_{1},t_{2}$) = 1 if $t_{\mathrm{msd},i}\leq T<t_{\mathrm{cf},i}$ and $I(t_{1},t_{2}$) = 0 otherwise, we obtain:

$p_{M,b}\left( T \right)=(1-\exp\left( -\beta_{M,b}{\times P}_{F}\left( T \right)\Delta t \right)I\left( t_{\mathrm{msd},i},t_{\mathrm{cf},i} \right),$ and

$$p_{F,b}\left( T \right)=(1-\exp\left( -\beta_{F,b}{\times P}_{M}\left( T \right)\Delta t \right)I\left( t_{\mathrm{fsd},i},t_{\mathrm{cf},i} \right).$$

These transition probabilities give the following Markov Chain for $T=t_{\mathrm{sd},i},\ldots,t_{\mathrm{cf},i}-\Delta t$,

$$s_{--,i}\left( T \right)=s_{--,i}\left( T-\Delta t \right){(1-p}_{M,b}\left( T \right))(1-p_{F,b}\left( T \right))$$

$$m_{b-, a1,i}\left( T \right)=s_{--,i}\left( T-\Delta t \right)p_{M,b}\left( T \right)\left( 1-p_{F,b}\left( T \right) \right)$$

$$m_{b-, a2,i}\left( T \right)=m_{b-, a1,i}\left( T-\Delta t \right)\left( 1-p_{F,b}\left( T \right) \right)$$

$$m_{b-,i}\left( T \right)=m_{b-,a2, i}\left( T-\Delta t \right)\left( 1-p_{F,b}\left( T \right) \right)+m_{b-, i}\left( T-\Delta t \right)\left( 1-p_{F,b}\left( T \right) \right)$$

$$f_{-b, a1,i}\left( T \right)=s_{--,i}\left( T-\Delta t \right)p_{F,b}\left( T \right)\left( 1-p_{M,b}\left( T \right) \right)$$

$$f_{-b, a2,i}\left( T \right)=f_{-b, a1,i}\left( T-\Delta t \right)\left( 1-p_{F,b}\left( T \right) \right)$$

$$f_{b-,i}\left( T \right)=f_{-b,a2, i}\left( T-\Delta t \right)\left( 1-p_{F,b}\left( T \right) \right)+f_{-b, i}\left( T-\Delta t \right)\left( 1-p_{F,b}\left( T \right) \right)$$

$$h_{b_{1}b_{2},i}\left( T \right)=h_{b_{1}b_{2},i}\left( T-\Delta t \right)+\left( \frac{p_{M,b}\left( T \right)}{p_{M,b}\left( T \right)+p_{F,b}\left( T \right)} \right)\left( s_{--,i}\left( T-\Delta t \right)p_{M,b}\left( T \right)p_{F,b}\left( T \right) \right)+\left( m_{b-,a1, i}\left( T-\Delta t \right)+m_{b-, a2,i}\left( T-\Delta t \right)+m_{b-, i}\left( T-\Delta t \right) \right)p_{F,b}\left( T \right)$$

$$h_{b_{2}b_{1},i}\left( T \right)=h_{b_{2}b_{1},i}\left( T-\Delta t \right)+\left( \frac{p_{F,b}\left( T \right)}{p_{M,b}\left( T \right)+p_{F,b}\left( T \right)} \right)\left( s_{--,i}\left( T-\Delta t \right)p_{M,b}\left( T \right)p_{F,b}\left( T \right) \right)+\left( f_{-b,a1, i}\left( T-\Delta t \right)+f_{-b,a2, i}\left( T-\Delta t \right)+f_{-b, i}(T-\Delta t) \right)p_{M,b}(T)$$

where the fraction is used to split up couples in which both partners were infected in the same month into the two different infection order states based on the competing risks of occurrence. The probabilities of being infected and surviving are simply the above infection probabilities multiplied by the probability of surviving the $t_{\mathrm{int},i}-T$ months until the DHS sampling for an individual aged $v_{M,i}(T)$ (where $v_{M,i}(T)$ is a function of $T$ because individuals’ ages are a function of time) the time of seroconversion which we estimate as described above and denote by $S(t_{\mathrm{int},i}-T,v_{M,i}(T))$,

${p'}_{M,b}\left( T \right)$=$p_{M,b}\left( T \right)S\left( t_{\mathrm{int},i}-T,v_{M,i}(T) \right), \mathrm{and}$

${p'}_{F,b}\left( T \right)$=$p_{F,b}\left( T \right)S\left( t_{\mathrm{int},i}-T,v_{F,i}(T) \right),$

where yielding the following iterative transition equations for the probabilities of being infected and also surviving until DHS sampling,

$${m'}_{b-, a1,i}\left( T \right)=s_{--,i}\left( T-\Delta t \right){p'}_{M,b}\left( T \right)\left( 1-p_{F,b}\left( T \right) \right)$$

$${m'}_{b-, a2,i}\left( T \right)={m'}_{b-, a1,i}\left( T-\Delta t \right)\left( 1-p_{F,b}\left( T \right) \right)$$

$${m'}_{b-, i}\left( T \right)={m'}_{b-, a2,i}\left( T-\Delta t \right)\left( 1-p_{F,b}\left( T \right) \right)$$

$${f^{'}}_{-b,a1, i}\left( T \right)=s_{--,i}\left( T-\Delta t \right){p^{'}}_{F,b}\left( T \right)\left( 1-p_{M,b}\left( T \right) \right)$$

$${f^{'}}_{-b,a2, i}\left( T \right)={f^{'}}_{-b, a1,i}\left( T-\Delta t \right)\left( 1-p_{M,b}\left( T \right) \right)$$

$${f'}_{-b, i}\left( T \right)={f'}_{-b, a2,i}\left( T-\Delta t \right)\left( 1-p_{M,b}\left( T \right) \right)$$

$${h'}_{b_{1}b_{2},i}\left( T \right)={h'}_{b_{1}b_{2},i}\left( T-\Delta t \right)+\left( \frac{{p'}_{M,b}\left( T \right)}{{p'}_{M,b}\left( T \right)+{p'}_{F,b}\left( T \right)} \right)\left( s_{--,i}\left( T-\Delta t \right){p'}_{M,b}\left( T \right){p'}_{F,b}\left( T \right) \right)+\left( {m'}_{b-,a1, i}\left( T-\Delta t \right)+{m'}_{b-,a2, i}\left( T-\Delta t \right)+{m'}_{b-, i}\left( T-\Delta t \right) \right){p'}_{F,b}\left( T \right)$$

$${h'}_{b_{2}b_{1},i}\left( T \right)={h'}_{b_{2}b_{1},i}\left( T-\Delta t \right)+\left( \frac{{p'}_{F,b}\left( T \right)}{{p'}_{M,b}\left( T \right)+p_{'F,b}\left( T \right)} \right)\left( s_{--,i}\left( T-\Delta t \right){p'}_{M,b}\left( T \right){p'}_{F,b}\left( T \right) \right)+\left( {f^{'}}_{-b, a1,i}\left( T-\Delta t \right)+{f^{'}}_{-b,a2, i}\left( T-\Delta t \right)+{f'}_{-b, i}(T-\Delta t) \right){p'}_{M,b}(T)$$

Infection during the partnership follows a similar iterative algorithm, though the forms of transmission are now from an HIV positive partner or from extra-couple intercourse with an individual of the opposite sex in the population at large. Because individuals infected in the last month prior to DHS sampling are unlikely to have yet seroconverted when sampled, we only iterate infections in couples up to the month prior to the interview, $T=t_{\mathrm{cf},i},\ldots,t_{\mathrm{fin},i}$, where $t_{\mathrm{fin},i}=t_{int,i}-\Delta t$. The respective probabilities of a male being infected by his female partner given she is HIV positive and vice versa are

$$p_{M,pa}=1-\exp\left( -{\alpha\beta}_{M,p}\Delta t \right), \mathrm{and}$$

$p_{F,pa}=1-\exp\left( -{\alpha\beta}_{F,p}\Delta t \right)$,

**during the acute phase, where** $\boldsymbol{\alpha}$ **is the relative risk of transmission from an individual in the acute vs chronic phase of HIV infection.** For the chronic phase,

$$p_{M,p}=1-\exp\left( -\beta_{M,p}\Delta t \right), \mathrm{and}$$

$p_{F,p}=1-\exp\left( -\beta_{F,p}\Delta t \right)$.

The corresponding joint probabilities of infection and survival to DHS sampling are, in contrast, dependent on timing and are respectively given by

${p'}_{M,pa}\left( T \right)$=$p_{M,pa}S\left( t_{\mathrm{int},i}-T,v_{M,i}(T) \right), \mathrm{and}$

${p'}_{F,pa}\left( T \right)$=$p_{F,pa}S\left( t_{\mathrm{int},i}-T,v_{F,i}(T) \right),$ **for the acute phase and**

${p'}_{M,p}\left( T \right)$=$p_{M,p}S\left( t_{\mathrm{int},i}-T,v_{M,i}(T) \right), \mathrm{and}$

${p'}_{F,p}\left( T \right)$=$p_{F,p}S\left( t_{\mathrm{int},i}-T,v_{F,i}(T) \right)$ for the chronic phase.

The respective probabilities of the male and female partner being infected during extra-couple intercourse in month $T=t_{\mathrm{cf},i},\ldots,t_{\mathrm{fin},i}$ vary with population prevalence in the opposite sex (as for transmission before couple formation above),

$$p_{M,e}\left( T \right)=1-\exp\left( -\beta_{M,e}{\times P}_{F}\left( T \right)\Delta t \right), \mathrm{and}$$

$$p_{F,e}\left( T \right)=1-\exp\left( -\beta_{F,e}{\times P}_{M}\left( T \right)\Delta t \right).$$

The corresponding joint probabilities of extra-couple infection in month $T$ with survival up until $t_{\mathrm{int},i}$ are then

${p'}_{M,e}\left( T \right)$=$p_{M,e}\left( T \right)S\left( t_{\mathrm{int},i}-T,v_{M,i}(T) \right), \mathrm{and}$

${p'}_{F,e}\left( T \right)$=$p_{F,e}\left( T \right)S(t_{\mathrm{int},i}-T,v_{F,i}(T))$.

We then can iterate couple serostatus probabilities with the following transition equations for $T=t_{\mathrm{cf},i},\ldots,t_{\mathrm{fin},i}$,

$$s_{--,i}\left( T \right)=s_{--,i}\left( T-\Delta t \right){(1-p}_{M,e}\left( T \right))(1-p_{F,e}\left( T \right))$$

$m_{b-,a1,i}\left( T \right)=0$ , because any male infected before couple formation will be in stage a2 by time $t_{cf}$.

$$m_{b-,a2,i}\left( T \right)=m_{b-,a1,i}\left( T-\Delta t \right){(1-p}_{F,pa})(1-p_{F,e}\left( T \right))$$

$$m_{b-,i}\left( T \right)=m_{b-,i}\left( T-\Delta t \right){(1-p}_{F,p})\left( 1-p_{F,e}\left( T \right) \right)+m_{b-,a2,i}\left( T-\Delta t \right){(1-p}_{F,pa})\left( 1-p_{F,e}\left( T \right) \right)$$

$$m_{e-,a1,i}\left( T \right)=s_{--,i}\left( T-\Delta t \right)p_{M,e}(T)(1-p_{F,e}(T))$$

$$m_{e-,a2,i}\left( T \right)=m_{e-,a1,i}\left( T-\Delta t \right){(1-p}_{F,pa})\left( 1-p_{F,e}\left( T \right) \right)$$

$$m_{e-,i}\left( T \right)=m_{e-,i}\left( T-\Delta t \right){(1-p}_{F,p})\left( 1-p_{F,e}\left( T \right) \right)+m_{e-,a2,i}\left( T-\Delta t \right){(1-p}_{F,pa})\left( 1-p_{F,e}\left( T \right) \right)$$

$f_{-b,a1,i}\left( T \right)=0$, because any female infected before couple formation will be in stage a2 by time $t_{cf}$.

$$f_{-b,a2,i}\left( T \right)=f_{-b,a1,i}\left( T-\Delta t \right){(1-p}_{M,pa})(1-p_{M,e}\left( T \right))$$

$$f_{-b,i}\left( T \right)=f_{-b,i}\left( T-\Delta t \right){(1-p}_{M,p})\left( 1-p_{M,e}\left( T \right) \right)+f_{-b,a2,i}\left( T-\Delta t \right){(1-p}_{M,pa})(1-p_{M,e}\left( T \right))$$

$$f_{-e,a1,i}\left( T \right)=s_{--,i}\left( T-\Delta t \right)p_{F,e}(T)(1-p_{M,e}(T))$$

$$f_{-e,a2,i}\left( T \right)=f_{-e,a1,i}\left( T-\Delta t \right){(1-p}_{M,pa})\left( 1-p_{M,e}\left( T \right) \right)$$

$$f_{-e,i}\left( T \right)=f_{-e,i}\left( T-\Delta t \right){(1-p}_{M,p})\left( 1-p_{M,e}\left( T \right) \right)+f_{-e,a2,i}\left( T-\Delta t \right){(1-p}_{M,pa})\left( 1-p_{M,e}\left( T \right) \right)$$

$$h_{b_{1}b_{2},i}\left( T \right)=h_{b_{1}b_{2},i}\left( T-\Delta t \right)$$

$$h_{b_{2}b_{1},i}\left( T \right)=h_{b_{2}b_{1},i}\left( T-\Delta t \right)$$

$$h_{be,i}\left( T \right)=h_{be,i}\left( T-\Delta t \right)+\left( m_{b-,a1,i}\left( T-\Delta t \right)+m_{b-,a2,i}\left( T-\Delta t \right) \right){\left( 1-p_{F,pa} \right)p}_{F,e}\left( T \right)+m_{b-,i}\left( T-\Delta t \right){(1-p_{F,p})p}_{F,e}(T)$$

$$h_{eb,i}\left( T \right)=h_{eb,i}\left( T-\Delta t \right)+\left( f_{-b,a1,i}\left( T-\Delta t \right)+f_{-b,a2,i}\left( T-\Delta t \right) \right){(1-p_{M,pa})p}_{M,e}(T)+f_{-b,i}\left( T-\Delta t \right){(1-p_{M,p})p}_{M,e}(T)$$

$$h_{bp,a,i}\left( T \right)=h_{bp,a,i}\left( T-\Delta t \right)+\left( m_{b-,a1,i}\left( T-\Delta t \right)+m_{b-,a2,i}\left( T-\Delta t \right) \right)p_{F,pa}$$

$$h_{bp,i}\left( T \right)=h_{bp,i}\left( T-\Delta t \right)+m_{b-,i}\left( T-\Delta t \right)p_{F,p}$$

$$h_{pb,a,i}\left( T \right)=h_{pb,a,i}\left( T-\Delta t \right)+\left( f_{-b,a1,i}\left( T-\Delta t \right)+f_{-b,a2,i}\left( T-\Delta t \right) \right)p_{M,pa}$$

$$h_{pb,i}\left( T \right)=h_{pb,i}\left( T-\Delta t \right)+f_{-b,i}\left( T-\Delta t \right)p_{M,p}$$

$$h_{ep,a,i}\left( T \right)=h_{ep,a,i}\left( T-\Delta t \right)+\left( m_{e-,a1,i}\left( T-\Delta t \right)+m_{e-,a2,i}\left( T-\Delta t \right) \right)p_{F,pa}$$

$$h_{ep,i}\left( T \right)=h_{ep,i}\left( T-\Delta t \right)+m_{e-,i}\left( T-\Delta t \right)p_{F,p}$$

$$h_{pe,a,i}\left( T \right)=h_{pe,a,i}\left( T-\Delta t \right)+\left( f_{-e,a1,i}\left( T-\Delta t \right)+f_{-e,a2,i}\left( T-\Delta t \right) \right)p_{M,pa}$$

$$h_{pe,i}\left( T \right)=h_{pe,i}\left( T-\Delta t \right)+f_{-e,i}\left( T-\Delta t \right)p_{M,p}$$

$$h_{e_{1}e_{2},i}\left( T \right)=h_{e_{1}e_{2},i}\left( T-\Delta t \right)+\left( \frac{p_{M,e}\left( T \right)}{p_{M,e}\left( T \right)+p_{F,e}\left( T \right)} \right)\left( s_{--,i}\left( T-\Delta t \right)p_{M,e}\left( T \right)p_{F,e}\left( T \right) \right)+\left( m_{e-,a1,i}\left( T-\Delta t \right)+m_{e-,a2,i}\left( T-\Delta t \right) \right)(1-p_{F,pa})p_{F,e}(T)+m_{e-,i}\left( T-\Delta t \right)(1-p_{F,p})p_{F,e}(T)$$

$$h_{e_{2}e_{1},i}\left( T \right)=h_{e_{1}e_{2},i}\left( T-\Delta t \right)+\left( \frac{p_{F,e}\left( T \right)}{p_{M,e}\left( T \right)+p_{F,e}\left( T \right)} \right)\left( s_{--,i}\left( T-\Delta t \right)p_{M,e}\left( T \right)p_{F,e}\left( T \right) \right)+\left( f_{-e,a1,i}\left( T-\Delta t \right)+f_{-e,a2,i}\left( T-\Delta t \right) \right)(1-p_{M,pa})p_{M,e}(T)+f_{-e,i}\left( T-\Delta t \right)(1-p_{M,p})p_{M,e}(T)$$

making the assumption that with each month, within-partnership transmission occurs before individuals have the opportunity for extra-couple transmission. The corresponding joint probabilities of a couple being in a serostatus group at month $T$ as well as being alive at the time of DHS sampling are given by

$${m'}_{b-,a1,i}\left( T \right)=0$$

$${m'}_{b-,a2,i}\left( T \right)={m'}_{b-,a1,i}\left( T-\Delta t \right){(1-p}_{F,pa})(1-p_{F,e}\left( T \right))$$

$${m'}_{b-,i}\left( T \right)={m'}_{b-,i}\left( T-\Delta t \right){(1-p}_{F,p})\left( 1-p_{F,e}\left( T \right) \right)+{m'}_{b-,a2,i}\left( T-\Delta t \right){(1-p}_{F,pa})(1-p_{F,e}\left( T \right))$$

$${m'}_{e-,a1,i}\left( T \right)=s_{--,i}\left( T-\Delta t \right){p^{'}}_{M,e}(T)(1-p_{F,e}(T))$$

$${m'}_{e-,a2,i}\left( T \right)={m^{'}}_{e-,a1,i}\left( T-\Delta t \right){(1-p}_{F,pa})\left( 1-p_{F,e}\left( T \right) \right)$$

$${m'}_{e-,i}\left( T \right)={m'}_{e-,i}\left( T-\Delta t \right){(1-p}_{F,p})\left( 1-p_{F,e}\left( T \right) \right)+{m'}_{e-,a2,i}\left( T-\Delta t \right){(1-p}_{F,pa})\left( 1-p_{F,e}\left( T \right) \right)$$

$${f'}_{-b,a1,i}\left( T \right)=0$$

$${f'}_{-b,a2,i}\left( T \right)={f'}_{-b,a1,i}\left( T-\Delta t \right){(1-p}_{M,pa})(1-p_{M,e}\left( T \right))$$

$${f'}_{-b,i}\left( T \right)={f'}_{-b,i}\left( T-\Delta t \right){(1-p}_{M,p})\left( 1-p_{M,e}\left( T \right) \right)+{f'}_{-b,a2,i}\left( T-\Delta t \right){(1-p}_{M,pa})(1-p_{M,e}\left( T \right))$$

$${f^{'}}_{-e,a1,i}\left( T \right)=s_{--,i}\left( T-\Delta t \right){p^{'}}_{F,e}(T)(1-p_{M,e}(T))$$

$${f^{'}}_{-e,a2,i}\left( T \right)={f^{'}}_{-e,a1,i}\left( T-\Delta t \right){(1-p}_{M,pa})\left( 1-p_{M,e}\left( T \right) \right)$$

$${f'}_{-e,i}\left( T \right)={f'}_{-e,i}\left( T-\Delta t \right){(1-p}_{M,p})\left( 1-p_{M,e}\left( T \right) \right)+{f'}_{-e,a2,i}\left( T-\Delta t \right){(1-p}_{M,pa})\left( 1-p_{M,e}\left( T \right) \right)$$

$${h'}_{b_{1}b_{2},i}\left( T \right)={h'}_{b_{1}b_{2},i}\left( T-\Delta t \right)$$

$${h'}_{b_{2}b_{1},i}\left( T \right)={h'}_{b_{2}b_{1},i}\left( T-\Delta t \right)$$

$${h'}_{be,i}\left( T \right)={h'}_{be,i}\left( T-\Delta t \right)+\left( {m^{'}}_{b-,a1,i}\left( T-\Delta t \right)+{m^{'}}_{b-,a2,i}\left( T-\Delta t \right) \right){\left( 1-p_{F,pa} \right)p^{'}}_{F,e}\left( T \right)+{m'}_{b-,i}\left( T-\Delta t \right){(1-p_{F,p})p'}_{F,e}(T)$$

$${h'}_{eb,i}\left( T \right)={h'}_{eb,i}\left( T-\Delta t \right)+\left( {f'}_{-b,a1,i}\left( T-\Delta t \right)+{f'}_{-b,a2,i}\left( T-\Delta t \right) \right){(1-p_{M,pa})p'}_{M,e}(T)+{f'}_{-b,i}\left( T-\Delta t \right){(1-p_{M,p})p'}_{M,e}(T)$$

$${h'}_{bp,a,i}\left( T \right)={h'}_{bp,a,i}\left( T-\Delta t \right)+\left( {m^{'}}_{b-,a1,i}\left( T-\Delta t \right)+{m^{'}}_{b-,a2,i}\left( T-\Delta t \right) \right){p'}_{F,pa}$$

$${h'}_{bp,i}\left( T \right)={h'}_{bp,i}\left( T-\Delta t \right)+{m'}_{b-,i}\left( T-\Delta t \right){p'}_{F,p}$$

$${h^{'}}_{pb,a,i}\left( T \right)={h^{'}}_{pb,a,i}\left( T-\Delta t \right)+\left( {f'}_{-b,a1,i}\left( T-\Delta t \right)+{f'}_{-b,a2,i}\left( T-\Delta t \right) \right){p^{'}}_{M,pa}$$

$${h'}_{pb,i}\left( T \right)={h'}_{pb,i}\left( T-\Delta t \right)+{f'}_{-b,i}\left( T-\Delta t \right){p'}_{M,p}$$

$${h'}_{ep,a,i}\left( T \right)={h'}_{ep,a,i}\left( T-\Delta t \right)+\left( {m^{'}}_{e-,a1,i}\left( T-\Delta t \right)+{m^{'}}_{e-,a2,i}\left( T-\Delta t \right) \right){p'}_{F,pa}$$

$${h'}_{ep,i}\left( T \right)={h'}_{ep,i}\left( T-\Delta t \right)+{m'}_{e-,i}\left( T-\Delta t \right){p'}_{F,p}$$

$${h'}_{pe,a,i}\left( T \right)={h'}_{pe,a,i}\left( T-\Delta t \right)+\left( {f'}_{-e,a1,i}\left( T-\Delta t \right)+{f'}_{-e,a2,i}\left( T-\Delta t \right) \right){p'}_{M,pa}$$

$${h'}_{pe,i}\left( T \right)={h'}_{pe,i}\left( T-\Delta t \right)+{f'}_{-e,i}\left( T-\Delta t \right){p'}_{M,p}$$

$${h^{'}}_{e_{1}e_{2},i}\left( T \right)={h^{'}}_{e_{1}e_{2},i}\left( T-\Delta t \right)+\left( \frac{{p^{'}}_{M,e}\left( T \right)}{{p^{'}}_{M,e}\left( T \right)+{p^{'}}_{F,e}\left( T \right)} \right)\left( s_{--,i}\left( T-\Delta t \right){p^{'}}_{M,e}\left( T \right){p^{'}}_{F,e}\left( T \right) \right)+\left( {m^{'}}_{e-,a1,i}\left( T-\Delta t \right)+{m^{'}}_{e-,a2,i}\left( T-\Delta t \right) \right)\left( 1-p_{F,pa} \right){p^{'}}_{F,e}\left( T \right)+{m'}_{e-,i}\left( T-\Delta t \right)(1-p_{F,p}){p'}_{F,e}(T)$$

$${h'}_{e_{2}e_{1},i}\left( T \right)={h'}_{e_{1}e_{2},i}\left( T-\Delta t \right)+\left( \frac{{p^{'}}_{F,e}\left( T \right)}{{p^{'}}_{M,e}\left( T \right)+{p^{'}}_{F,e}\left( T \right)} \right)\left( s_{--,i}\left( T-\Delta t \right){p^{'}}_{M,e}\left( T \right){p^{'}}_{F,e}\left( T \right) \right)+\left( {f'}_{-e,a1,i}\left( T-\Delta t \right)+{f'}_{-e,a2,i}\left( T-\Delta t \right) \right)(1-p_{M,pa}){p'}_{M,e}(T)+{f'}_{-e,i}\left( T-\Delta t \right)(1-p_{M,p}){p'}_{M,e}(T)$$

The probability that a couple is then observed at month $t_{\mathrm{int},i}$ with any of the four serostatuses is then given by

$y_{i} \sim Multinomial\left( \frac{s_{i}\left( t_{\mathrm{fin},i} \right)}{n_{i}^{'}\left( t_{\mathrm{fin},i} \right)},\frac{m_{i}^{'}\left( t_{\mathrm{fin},i} \right)}{n_{i}^{'}\left( t_{\mathrm{fin},i} \right)},\frac{f_{i}^{'}\left( t_{\mathrm{fin},i} \right)}{n_{i}^{'}\left( t_{\mathrm{fin},i} \right)},\frac{h_{i}^{'}\left( t_{\mathrm{fin},i} \right)}{n_{i}^{'}\left( t_{\mathrm{fin},i} \right)} \right),$

where $y_{i}$ is the observed serostatus for the $i$-th couple and

$n_{i}^{'}\left( t_{\mathrm{fin},i} \right)=s_{i}\left( t_{\mathrm{fin},i} \right)+m_{i}^{'}\left( t_{\mathrm{fin},i} \right)+ f_{i}^{'}\left( t_{\mathrm{fin},i} \right)+h_{i}^{'}\left( t_{\mathrm{fin},i} \right)$.

We use infection status at time $t_{\mathrm{int},i}-\Delta t$ to indicate an approximately one month lag between infection and seroconversion (i.e., individuals infected less than a month before being tested are unlikely to test positive). We can explore how ignoring AIDS deaths biases the analysis by using the marginal serostatus probabilities,

$$y_{i} \sim Multinomial\left( s_{i}\left( t_{\mathrm{fin},i} \right),m_{i}\left( t_{\mathrm{fin},i} \right),f_{i}\left( t_{\mathrm{fin},i} \right),h_{i}\left( t_{\mathrm{fin},i} \right) \right),$$

and comparing the results. These probability distributions account only for sampling stochasticity: that is, we ignore demographic stochasticity, an assumption that holds well for large population sizes.

**3c. Proportion of observed infections from each transmission route**

Aside from estimating the six transmission coefficients $\left\{ \beta_{M,b},\beta_{M,p},\beta_{M,e},\beta_{F,b},\beta_{F,p},\beta_{F,e} \right\}$, we would also like to know the probability that a given observed infection occurred through each of the three possible routes (before partnership, from partner during partnership, extra-couply during partnership). When we do this, we can condition on survival and serostatus. For instance, the probability a male-positive serodiscordant couple sampled by DHS was infected by extra-couple transmission is

$${\pi'}_{e-,i}=\frac{{m^{'}}_{e-,*,i}(t_{\mathrm{fin},i})}{{m^{'}}_{i}(t_{\mathrm{fin},i})},$$

where $\pi'$ are used more generally to give the probability of the specified route of transmission (e-), survival given the serostatus group (+-) and survival, and ‘*’ denotes aggregation of both the acute and chronic phase state probabilities. Thus, the probability a male in a concordant positive couple sampled by DHS was infected extra-couply is

$${\pi'}_{e+,i}=\frac{{h^{'}}_{eb,i}\left( t_{\mathrm{fin},i} \right)+{h^{'}}_{ep,*,i}\left( t_{\mathrm{fin},i} \right)+{h^{'}}_{ee,i}(t_{\mathrm{fin},i})}{{h^{'}}_{i}\left( t_{\mathrm{fin},i} \right)}.$$

Finally, as a last example, the probability a female in a concordant positive couple sampled by DHS was infected by her male partner is

${\pi'}_{+p,i}=\frac{{h^{'}}_{bp,*,i}\left( t_{\mathrm{fin},i} \right)+{h^{'}}_{ep,*,i}\left( t_{\mathrm{fin},i} \right)}{{h^{'}}_{i}\left( t_{\mathrm{fin},i} \right)}$,

**and the probability she was infected during his acute phase is,**

${\pi'}_{+p,a,i}=\frac{{h^{'}}_{bp,a,i}\left( t_{\mathrm{fin},i} \right)+{h^{'}}_{ep,a,i}\left( t_{\mathrm{fin},i} \right)}{{h^{'}}_{i}\left( t_{\mathrm{fin},i} \right)}$.

We can estimate the proportion of all observed concordant positive couples in which the female was infected by her partner by simply averaging this quantity across couples,

$${\pi'}_{+p}=\frac{\sum{\pi'}_{+p,*,i}}{K_{h}} for each ith couple that is concordant positive$$

where $K_{h}$ is the number of such couples in the data set.

**3d. Proportion of infections from each transmission route inferred to the greater population**

**Same as previous section 3d but with minor notational errata corrected.**

While the above $\pi'$’s give us the probability of specified transmission routes for observed couples, they do not account for survival bias (i.e., the couples that are unobserved due to death which are systematically different from live couples). We take an inclusion probability approach to extrapolate to unobserved couples. This approach is best illustrated with an example followed by a more general, rigorous mathematical description.

As an example, imagine we observe one HIV positive man in a random sample. Imagine we also know that (based on his date of infection and studies of HIV survival times) he had only had a 50% chance of having survived up to the date we saw him. Then we can state that though we only found one infected man in this study, we know that other infected men may have died. We thus extrapolate to a population not biased by AIDS mortality and estimate that this one infected man represents 1/0.5 = 2 infected men in such a population. Now, imagine we also know that the probabilities his infection occurred pre-couply, extra-couply, or within-couple are 60%, 30%, and 10%, respectively, and given each of those transmission routes the probability he survived was 0.2, 0.4, and 0.3, respectively (survival probabilities differ because the timing of seroconversion depends on the route). Then we can similarly extrapolate to a population not biased by AIDS survival: we estimate the total number of pre-couple infections this man represents as 0.6/0.2 = 3.

Generally speaking, if we estimate that an event $X$ occurred with probability $p_{X}$ and we had probability $d_{X}$ of detecting it, we estimate there were $n_{X}=p_{X}/d_{X}$ such events $X$ in total. If we estimate that event $X$ occurred with probability $p_{X}$ but it could have occurred in one of two ways, $X_{1}$ or $X_{2},$ each with a distinct probability of having happened, ${p_{X}}_{1}$ and ${p_{X}}_{2}$ such that $p_{X}={p_{X}}_{1}+{p_{X}}_{2}$, and a distinct probability of detection, ${d_{X}}_{1}$ and ${d_{X}}_{2}$, then we estimate the total number of events $X_{1}$ and $X_{2}$ represented by the observed event $X$ as

$n_{X_{1}}=\frac{{p_{X}}_{1}}{{p_{X}}_{1}+{p_{X}}_{2}}\times\left( \frac{{p_{X}}_{1}{d_{X}}_{1}+{p_{X}}_{2}{d_{X}}_{2}}{{p_{X}}_{1}+{p_{X}}_{2}} \right)^{-1},$

$n_{X_{2}}=\frac{{p_{X}}_{2}}{{p_{X}}_{1}+{p_{X}}_{2}}\times\left( \frac{{p_{X}}_{1}{d_{X}}_{1}+{p_{X}}_{2}{d_{X}}_{2}}{{p_{X}}_{1}+{p_{X}}_{2}} \right)^{-1},$

where the first term in each equation is the probability $X_{1}$ or $X_{2}$, respectively, occurred given $X$ occurred, and the second term is the probability of detecting any event of type $X$ given that it occurred and its inverse can be considered an inflation factor that extrapolates to the total number of such events that we think occurred for every event $X$ observed. Scaling up to multiple events occurs similarly. If we observed ten such events $X$, each with probability ${p_{X}}_{i}={p_{X}}_{1,i}+{p_{X}}_{2,i}$ of occurring for $i=1,\ldots,10$, and probabilities ${d_{X}}_{1,i}$ and ${d_{X}}_{2,i}$ of being detected given that $X$ occurred as $X_{1}$ or $X_{2}$, respectively, then the total number of events $X_{1}$ and $X_{2}$, that we think are represented by our observations are

$${N_{X}}_{1}=\sum_{i=1}^{10} \frac{{p_{X}}_{1,i}}{{p_{X}}_{1,i}+{p_{X}}_{2,i}}\times\left( \frac{{p_{X}}_{1,i}{d_{X}}_{1,i}+{p_{X}}_{2,i}{d_{X}}_{2,i}}{{p_{X}}_{1,i}+{p_{X}}_{2,i}} \right)^{-1}, \mathrm{and}$$

$${N_{X}}_{2}=\sum_{i=1}^{10} \frac{{p_{X}}_{2,i}}{{p_{X}}_{1,i}+{p_{X}}_{2,i}}\times\left( \frac{{p_{X}}_{1,i}{d_{X}}_{1,i}+{p_{X}}_{2,i}{d_{X}}_{2,i}}{{p_{X}}_{1,i}+{p_{X}}_{2,i}} \right)^{-1}.$$

These can be simplified to

$${N_{X}}_{1}=\sum_{i=1}^{10} \frac{{p_{X}}_{1,i}}{{p_{X}}_{1,i}{d_{X}}_{1,i}+{p_{X}}_{2,i}{d_{X}}_{2,i}},$$

which is also equivalent to

$${N_{X}}_{1}=\sum_{i=1}^{10} \frac{{p_{X}}_{1,i}d_{X_{1},i}}{{p_{X}}_{1,i}{d_{X}}_{1,i}+{p_{X}}_{2,i}{d_{X}}_{2,i}}\times\left( {d_{X}}_{1,i} \right)^{-1},$$

where the latter form can be seen as the conditional probability of $X_{1}$ having occurred and being detected given $X$ occurred and was detected, inflated by the detection probability of event $X_{1}$ given it occurred. We hereafter use the shortest version, though all are equivalent.

The proportion of times that event $X$ occurred as $X_{1}$ or $X_{2}$, respectively, in the total (observed & unobserved) population is then estimated as

$${\pi_{X}}_{1}=\frac{{N_{X}}_{1}}{{N_{X}}_{1}+{N_{X}}_{2}}, \mathrm{and}$$

$${\pi_{X}}_{2}=\frac{{N_{X}}_{2}}{{N_{X}}_{1}+{N_{X}}_{2}}.$$

We now apply these principles to our model where the probability of detection is equivalent to the probability of survival-to-sampling in this framework. First we define the sets $S,M,F, \mathrm{and}H$ to include all values of $i$ for which the $i$-th couple is concordant negative, male-positive serodiscordant, female-positive serodiscordant, and positive concordant, respectively. If we want to estimate the proportion of all seropositive males in serodiscordant couples that were infected via extra-couple transmission unconditional on both partners’ survival to sampling, we have

$$\pi_{e-}=\frac{\sum_{i\in M} \frac{m_{e-,*,i}(t_{\mathrm{fin},i})}{{m^{'}}_{b-,*,i}(t_{\mathrm{fin},i})+{m^{'}}_{e-,*,i}(t_{\mathrm{fin},i})}}{\sum_{i\in M} \frac{m_{b-,*,i}(t_{\mathrm{fin},i})}{{m^{'}}_{b-,*,i}(t_{\mathrm{fin},i})+{m^{'}}_{e-,*,i}(t_{\mathrm{fin},i})}+\sum_{i\in M} \frac{m_{e-,*,i}(t_{\mathrm{fin},i})}{{m^{'}}_{b-,*,i}(t_{\mathrm{fin},i})+{m^{'}}_{e-,*,i}(t_{\mathrm{fin},i})}},$$

where summations occur over all couples $i$ that are male-positive serodiscordant. This simplifies to

$$\pi_{e-}=\frac{\sum_{i\in M} \frac{m_{e-,*,i}\left( t_{\mathrm{fin},i} \right)}{{m^{'}}_{i}\left( t_{\mathrm{fin},i} \right)}}{\sum_{i\in M} \frac{m_{b-,*,i}\left( t_{\mathrm{fin},i} \right)}{{m^{'}}_{*,i}\left( t_{\mathrm{fin},i} \right)}+\sum_{i\in M} \frac{m_{e-,*,i}\left( t_{\mathrm{fin},i} \right)}{{m^{'}}_{*,i}\left( t_{\mathrm{fin},i} \right)}},$$

$$and finally to$$

$$\pi_{e-}=\frac{\sum_{i\in M} \frac{m_{e-,*,i}(t_{\mathrm{fin},i})}{{m^{'}}_{i}(t_{\mathrm{fin},i})}}{\sum_{i\in M} \frac{m_{i}(t_{\mathrm{fin},i})}{{m^{'}}_{i}(t_{\mathrm{fin},i})}}.$$

At this point, however, we remark that while we do account for AIDS deaths, our model implicitly has seropositive individuals infecting their partners after they die. Modelling death times accurately in this Markovian framework is not possible because we would have to track both individuals’ time since infection for each potential month they get infected in, increasing the number of state variables to unreasonable numbers. Even with this problem, however, our model state probabilities for couples being in each of the live states are still correct and, thus, this does not impact or bias our ability to fit data on live couples (${{s_{i}^{'}\left( t_{fin,i} \right),h}^{'}}_{i}\left( t_{fin,i} \right),$ ${m'}_{i}(t_{fin,i})$ or ${f'}_{i}(t_{fin,i})$). However, this limits our ability to infer transmission routes for unobserved couples because some of the probability that is in $h_{i}(t_{fin,i})$ should actually be in $m_{i}(t_{fin,i})$ or $f_{i}(t_{fin,i})$. Thus, the above expression for $\pi_{e-}$is not accurate. This is only a problem for the second infection in a couple, however, because the first infection can never have occurred after an AIDS death of a partner. Thus, we restrict our estimates of the proportion of transmission by route only to index infections within couples. In other words, we ask what transmission routes are responsible for first introducing HIV into a previously concordant negative couple. For instance, the proportion of all index infections that were extra-couple infections of males can be estimated by either

$${\pi^{G}}_{e_{1}.}=\frac{\sum_{i\in M,F,H} \frac{m_{e-,*,i}\left( t_{\mathrm{fin},i} \right)+h_{e_{1}e_{2},i}(t_{\mathrm{fin},i})+h_{ep,*,i}(t_{\mathrm{fin},i})}{{m^{'}}_{i}\left( t_{\mathrm{fin},i} \right)+{f^{'}}_{i}\left( t_{\mathrm{fin},i} \right)+h_{i}^{'}(t_{\mathrm{fin},i})}}{\sum_{i\in M,F,H} \frac{m_{i}\left( t_{\mathrm{fin},i} \right)+f_{i}\left( t_{\mathrm{fin},i} \right)+h_{i}(t_{\mathrm{fin},i})}{{m^{'}}_{i}\left( t_{\mathrm{fin},i} \right)+{f^{'}}_{i}\left( t_{\mathrm{fin},i} \right)+h_{i}^{'}(t_{\mathrm{fin},i})}}$$

or

$${\pi^{G}}_{e_{1}.}=\frac{\sum_{\forall i} \frac{m_{e-,*,i}\left( t_{\mathrm{fin},i} \right)+h_{e_{1}e_{2},i}(t_{\mathrm{fin},i})+h_{ep,*,i}(t_{\mathrm{fin},i})}{s_{i}\left( t_{fin,i} \right)+{m^{'}}_{i}\left( t_{\mathrm{fin},i} \right)+{f^{'}}_{i}\left( t_{\mathrm{fin},i} \right)+h_{i}^{'}(t_{\mathrm{fin},i})}}{\sum_{\forall i} \frac{m_{i}\left( t_{\mathrm{fin},i} \right)+f_{i}\left( t_{\mathrm{fin},i} \right)+h_{i}(t_{\mathrm{fin},i})}{{s_{i}\left( t_{fin,i} \right)+m^{'}}_{i}\left( t_{\mathrm{fin},i} \right)+{f^{'}}_{i}\left( t_{\mathrm{fin},i} \right)+h_{i}^{'}(t_{\mathrm{fin},i})}},$$

where the numerator is the number of inflated male-index infections and the denominator is the total number of inflated index infections. In the former case, only infected couples are used in the estimator, while the latter uses all couples. Given the complexity of these estimators, we use a simulation approach in which the proportion of transmission occurring from each route is known even amongst couples in which one or more partner dies prior to DHS sampling. We compared the above estimators (which only use data from couples where both partners live to DHS sampling) of their respective true values for 100 simulations. We chose the second estimator due to its lower bias and variance (Table S3).

In summary, we track the following proportions (unconditional on survival) the proportion of all index infections in couples that were male or female infections before couple formation (${\pi^{G}}_{b_{1}.}, {\pi^{G}}_{.b_{1}}$) and via extra-couple transmission (${\pi^{G}}_{e_{1}.}, {\pi^{G}}_{.e_{1}}$).

**3e. Projecting future incidence over the next year**

See previous section **3e**.

**3f. Model Fitting**

Identical to previous section **3f** with the following exceptions:

**4. Sensitivity analyses**

Table S2 describes our model assumptions, their justifications, and their implications in tabular form. We conducted sensitivity analyses to several of these assumptions as outlined below.

**4a. Heterogeneity**

Same as previous section **4a** with the additional sensitivity analysis to heterogeneity in within-couple transmission as well as assortativity in this heterogeneity (previous work only considered the effects of pre- and extra-couple heterogeneity on models fitted with homogenous parameters and did not consider assortativity).

**4b-e.**

Unchanged from sections **4b-4e** (sensitivity analyses to couples excluded by the inclusion criteria, ART coverage assumptions, and reporting bias of female sexual debuts) in the previous online web appendix.

**5. Simulation Model**

The conceptual framework of the simulation model used in this manuscript is similar to that published previously,^1^ but differs in that—instead of modeling each couple is having a probability of being in each serostatus type during every month of their sexually active lifetime (i.e. a Markov chain state probability model)—transmission is modeled as a discrete process with HIV infection, AIDS mortality, and couples aging out of the cohort occurring as discrete events in time. This allows us to better capture the proportion of the couple population in any given serostatus and how that changes over time in order to better understand what drives the serodiscordance proportion.

**5a. Event-driven algorithm**

HIV transmission was modeled independently for each couple (or pseudocouple, see next sections) in each simulation as follows. Male and female partners were both at risk for transmission from HIV before couple formation starting in the month of their sexual debuts up until the month before they joined into a couple. For each individual, their probability of infection in each month was calculated as described in section **3b**. Any partners infected pre-couple formation are assigned date of death from AIDS mortality by sampling a random variable from the Weibull distribution corresponding to the age-at-seroconversion dependent survival functions described in section **2c**. Any couples for which one or more partners is infected pre-couple formation and assigned to die of AIDS pre-couple formation are immediately discarded from the simulation (as they would never become a couple and therefore are not relevant to couple simulations).

All other couples next proceed to the couple phase of the simulation model. The probability of infection via extra- or within-couple transmission in each month is calculated as in section **3b**, with the exception that because partner serostatuses are known to be seronegative or seropositive (rather than being probabilistically determined) at a given time, within-couple transmission occurs conditional on having a seropositive partner. As above, once a partner is infected either extra- or within-couply, they are assigned a date of death due to AIDS mortality. Each couple is kept as alive and sample-able (per DHS) in the simulation model up until (1) the first partner dies of AIDS or (2) the first partner ages out of the DHS cohort (>50 yr for women, > 60 yr for men). Simulations continued up until 2011 (i.e., the latest UNAIDS country-wide prevalence estimates available at the time of this analysis).

Acute phase transmission was modeled as above in section **3b** with seronegative partners at increased risk from their recently infected partners for 2 months after the latter’s month of infection. We conducted simulations over a range of acute to chronic relative hazards (1-50) and used transmission coefficients estimated from fitting the model in **3b** with the same relative hazard (i.e. simulations with acute phase relative hazard of 5 used transmission coefficients fit with a relative hazard of 5).

Heterogeneity was added as described in section **3b**. Each individual was given a log-normally distributed random ‘risk’ deviate determining their susceptibility to infection relative to the population mean. Lognormal distributions were centered at $\mu=-\frac{\sigma^{2}}{2}$ to ensure that risk was centered around the population mean of homogenous simulations (i.e. accounting for the fact that the lognormal distribution’s mean is $exp(\mu+\frac{\sigma^{2}}{2})$). Assortativity was modeled by drawing these lognormal risk factors from bivariate correlated distributions with correlation $\rho$ on the lognormal scale.

**5b. Couple Pseudo-Populations**

We developed methods to synthesize populations of couples (i.e. pseudo-populations) rather than use the empirical DHS sample populations in our simulations for two different reasons. Firstly, DHS sample sizes vary widely between countries with some of them well under a thousand. Given that we want to understand SDP dynamics at a large scale and not worry about sampling variability and demographic stochasticity due to small sample size, we wanted to be able to simulate transmission in much larger couple populations. Secondly, we wanted to account for the survival bias inherent in cross-sectional data sets such as DHS (i.e., the only couples that can be sampled by such surveys are those for which both members are alive at the time of sampling). Without accounting for this bias, we would be initializing simulations at the beginning of the HIV epidemic in the sub-population of couples that lived until a recent DHS survey. We created two options for creating pseudo-populations. The first is based on pseudo-replicating observed couples based on their inverse probability of surviving to DHS sampling. The second extracts a multivariate signature of the five key relationship variables for each couple (ages at sexual debuts for men and women, time between sexual debut and couple formation for each gender, and date of couple formation) for each country using Gaussian copula models and then uses these same models to randomly generate new couples with similar relationship patterns. The final results shown use the parametric approach though all results were robust to choice of couple simulator.

**5b. i. Non-Parametric Pseudo-Replication Approach**

In the non-parametric approach, pseudo-populations were constructed by calculating the probability that couples observed in the DHS survived to their interview time (see section **3b**). The inverse of these probabilities was then use to weight the occurrence of couples in the pseudo-population. To reduce the variation in results caused by demographic stochasticity, we increased the size of the pseudo-populations relative the size of the DHS sample populations by replicating each observed couple $X_{i}$ times where $X_{i} \sim Poisson(\frac{S}{Z_{i}})$, $Z_{i}$ is the probability that both members of the *i*-th couple survived until their DHS interview, and S is a scalar that increases the number of couples such that a much larger population can be created. We choose S such that the resulting population is well over 100,000 and then randomly sample 100,000 pseudo-couples from that population to yield our final representative pseudopopulation. The advantages of this approach are that we do not have to make any assumptions about the correlational structure of males’ and females’ sexual debuts and their date of couple formation.

The disadvantage of this approach arises because in some cases the transmission parameters were fit to countries for which there were greater than one DHS surveys performed at different times. In such cases, in our simulations at the time of the earlier DHS surveys the pseudo-population reflects couples that were observed at later interviews but not the current one. For instance, for a country in which DHS surveys were done in both 2003 and 2011 the pseudo-population is a combination of all couples that would have been available at the 2003 interview to be sampled had there not been AIDS mortality and the same for those that would have been available at the 2011 interview. A second problem with this approach is that when we look at the simulated time series the pseudo-population is missing couples that would’ve been too old to be interviewed in the DHS survey. For instance, if a country had a single survey done in 2010 then the pseudo-population examined in 1995 include only those couples who would have been available to be sampled in 2010. So couples for which the male and female partners were, respectively, over the ages of 59 or 49 years by 2010 would not be included in the simulation even in earlier times. The following approach avoids these issues.

**5b. ii. Parametric Distributional Approach**

As a second alternative approach we developed Gaussian copula models of relationship variables to extract sexual life history ‘signatures’ specific to each country and then use these models to randomly generate couples representative of each country’s ‘signature’. Both our probability state space model used to fit the DHS data and our event-driven simulation model rely on five relationship history variables for each couple: (1) male and (2) female ages at sexual debut (first time having intercourse); time duration between sexual debut and couple formation for (3) male and (4) female partners; and (5) calendar date of couple formation. The distribution of these five variables exhibits nontrivial correlations within each country-group data set, and their multivariate distributions differ greatly between countries.

To generate couples with relationship histories representative of observed couples we fit Gaussian copula models in the following way for each country-group.

1. Fit a continuous kernel density function to the empirical distribution of each of the five relationship variables using either the ‘density’ or ‘logspline’ functions in R weighting each couple by their inverse probability of surviving to be sampled in the DHS and choosing the smoothing bandwidth empirically based on visual inspection of the fit to histograms.
2. Discretize the fitted density function into monthly intervals to create a probability distribution function (PDF) for each of the five random variables. The resulting PDF for age at male sexual, for example, could then generate random ages at male sexual debut representing the fitted country group. However, these five PDFs are still independent and in the next steps multivariate correlation structures are fitted.
3. Transform these PDFs into cumulative density functions (CDFs).
4. Use these CDFs to transform the observations of these five variables for each couple to their quantile on [0,1].
5. Use the normal quantile function ‘qnorm’ to transform each of these quantiles to their corresponding value on (-∞, ∞).
6. Use ‘cov.wt’ to get the covariance-variance matrix for these five variables where each couple is weighted by their inverse probability of surviving to be observed in the DHS sample.

Then, to generate 100,000 couples with the above covariance-variance structure:

1. Generate 100,000 correlated multivariate normal vector of dimension five with the above covariance-variance structure.
2. Transform these variables to their quantiles on [0, 1] using the normal CDF (‘pnorm’).
3. Use the inverse CDFs created in step (3) above to transform these correlated quantile vectors back to discrete monthly vectors with the observed correlation structure.

Figure S2 compares pairwise plots for the five relationship variables both for the observed data and fitted copula model.

**6. Counterfactual Simulations**

To understand which processes affect the serodiscordant proportion (SDP) we used the event-driven model (**5a**) to simulate epidemics in couple pseudo-populations (**5b**) both ‘as observed’—i.e. with the transmission coefficients as fitted to the DHS data (**3a-f**)—as well as counterfactual epidemics with various processes altered to understand how they affect the SDP trajectory. The first counterfactual scenario removed AIDS mortality from the simulation such that couples only are removed from the simulation when a partner ages out of the DHS age limitations (>50 for women, >60 for men). The next set of counterfactual scenarios simulated epidemics with the intrinsic transmission rate or the pre- or extra-couple contact coefficients scaled to either 0, 0.1, 0.2, 0.5, 2, 5, or 10 times their fitted value (except for the intrinsic transmission rate which was not scaled to 0 since that resulted in no epidemic). In each counterfactual scenario, we always applied the same scalar to both male and female parameters.

Next we simulated counterfactual epidemics as fitted but with individual heterogeneity in transmission. We allowed for individuals to have random log-normally-distributed transmission risk factors that increased or decreased their susceptibility to infection by affecting their baseline transmission rate, ($\beta_{M}^{*}$ or $\beta_{F}^{*}$) . We also allowed for heterogeneity in sexual network characteristics by conducting simulations where either pre- or extra-couple contact coefficients were heterogeneous. Importantly, these counterfactual simulations are not parameterized by estimates of the variance made from real DHS data (which is computationally challenging) but rather rely on the addition of heterogeneity to parameterizations fitted to the real data with a homogenous model. Nevertheless, this approach helps elucidate how heterogeneity in each transmission route affects the SDP.

After noting that SDP is only sensitive to heterogeneity in the baseline transmission rates ($\beta_{M}^{*}$ and $\beta_{F}^{*}$) and not pre- or extra-couple contact coefficients ($c_{M, p}$, $c_{M,e}$,$c_{F, p}$, $c_{F,e}$), we reran all homogenous simulations (as observed, no AIDS mortality, and scaled transmission routes) with heterogeneity in the baseline transmission rates to explore the robustness of these results. In these simulations, we used log-normally distributed risk factors with standard deviations of 0.5, 1, 2, or 3, and considered assortativity between partners in these risk factors of $\rho$ = 0, 0.2, .4, and 0.8.

**7. Couple Dissolution Analysis**

Our couple-centric model in both its state probability (**3**) and event-driven (**5**) forms does not include couple dissolution as a process and thus makes the assumption that the couples only form and do not dissolve (i.e. disappear from the couple population base as sampled by DHS). We make this assumption because we cannot match partners from dissolved couples in the DHS survey (since the survey is household-based, and partners from dissolved couples no longer cohabit) and therefore never have relationship history and serostatus data from partners who were formerly together in a stable couple. Furthermore, there exist no reliable estimates of couple duration expectancy distributions for the countries analyzed. We do not however expect this assumption to have biased our transmission coefficient estimates because the majority of couples analyzed were in their first cohabiting relationship both because a large proportion of all couples identified cross-sectionally were in their first cohabitation and because we had to remove couples for which neither partner was in their first cohabitation since data on their relationship duration was missing (see **2a**).

Nevertheless, couple dissolution rates could affect SDP and potentially explain differences between countries. If the distribution of couple duration expectancies (i.e. up until dissolution) differed dramatically between countries then we might expect countries with much shorter durations to exhibit greater SDPs because concordant positivity is always the last serostatus in all possible serostatus orders (i.e. it follows serodiscordance usually) and therefore curtailing couple durations will reduce couple-time spent concordant positive. Furthermore, partners from concordant positive couples are more likely to reform into two serodiscordant couples when reforming new couples

(as long as population wide prevalence is < 0.5), further increasing the SDP.

We examined the hypothesis that between-country variation in SDP is driven by differences in couple dissolution rates by assessing whether the mean relationship duration in DHS couples negatively correlates with SDP using a univariate normal regression. We performed this regression in each of the following ways: (1) with the raw DHS data; (2) weighting the couples by their inverse probability of survival; (3) and (4) are the same as (1) and (2) except only couples with both partners in their first partnership were analyzed to remove the effect of individuals from dissolved concordant positive couples forming new relationships.

Between-country variability in SDP did not significantly correlate with differences in couple dissolution rates as measured by average relationship durations. Regressions of SDP against average relationship duration yielded positive but statistically insignificant trends (P > 0.05; Figure S3) regardless of whether all couples were analyzed as is (slope of 0.0793; 95% CI -0.0843 to 0.103) or after accounting for AIDS-survival bias (0.0276; 95% CI -0.0757 to 0.131) or whether only couples with both partners in their first partnership were analyzed (as is 0.0406; 95% CI -0.0406 to 0.168; accounting for AIDS-survival bias: 0.0793; 95% CI -0.0499 to 0.209).

**8. Drivers of Between-country Variation in Prevalence**

Logistic prevalence—measured as peak epidemic prevalence, or prevalence amongst individuals in couples in the DHS sample under consideration—was regressed against candidate models include some or all of the following parameters—each on a log scale: the geometric average of gender-specific within-couple transmission rates ($\beta^{*}=\sqrt{\beta_{M}^{*}\times\beta_{F}^{*}})$, the geometric average of gender-specific extra-couple mixing coefficients ($c_{\mathrm{extra}}^{*}=\sqrt{c_{M,e}^{*}\times c_{F,e}^{*}}$), and male and female pre-couple sexual mixing coefficients ($c_{M,b}$, $c_{F,b}$).

We used the geometric averages for within- and extra-couple parameters because their values did not differ substantially between genders for all countries examined, and by taking the geometric averages from our Bayesian fits we could substantially reduce the variance of these estimates as well as reduce collinearity between the explanatory variables in the regression. In contrast, women and girls were consistently at much greater transmission risk compared to men and boys pre-couple formation so we kept these parameters as gender-specific.

To account for the uncertainty in the estimates of the independent variables in these regressions we weighted each country by the inverse variance of the estimate in univariate regressions. In multivariate regressions, there were multiple independent variables each with their own estimates of uncertainty. However, variance estimates were highly correlated amongst explanatory variables—countries with greater sample sizes and HIV prevalence had more precision in all parameter estimates—so we reran multivariate regressions with weights from each of the four different independent variables for robustness. The choice of variance used to weight each country had negligible quantitative and no qualitative impact on regression results. Our final models as presented in the main text are weighted using the inverse geometric average of the variances of all explanatory variables.

**9. Reproduction of results and extension to the model**

To increase the transparency of our methods and encourage the use of our modeling paradigm to understand cross-sectional survey data, we have provided the cleaned data sets necessary to replicate this work in an online supporting information file along with the R scripts needed to fit or simulate our cople transmission model. Any research relying on the DHS data provided here (or on the DHS website) *must be first registered on the DHS website, must send DHS copies of the publication, and must properly credit DHS* (<http://www.measuredhs.com/>).

**10. Online Supplementary Files**

All scripts and data sets necessary to reproduce our results are provided in the files listed below.

**File S5 – Bellan R data files.zip:** ZIP archive containing *.Rdata files and *.R scripts used to perform analyses on a supercomputing cluster. Scripts include substantial comments but please feel free to contact authors if you are interested in learning more about the organization of the analysis.

NOTE: The Ugandan 2004-2005 AIDS Indicator Survey is a restricted DHS data set not normally available for analysis. We received permission to include this data set in our analysis from the Ugandan Ministry of Health. For that reason, we have not included that data in the above file.

**B. Cited References**

1. Bellan, S. E. *et al.* Extra-couple HIV transmission in sub-Saharan Africa: a mathematical modelling study of survey data. *Lancet* **6736,** 1–9 (2013).

2. UNAIDS. *Global report: UNAIDS report on the global AIDS epidemic*. (UNAIDS, 2010).

3. Cohen, M. S., Shaw, G. M., McMichael, A. J. & Haynes, B. F. Acute HIV-1 Infection. *N Engl J Med* **364,** 1943–1954 (2011).

4. Hethcote, H. W. The mathematics of infectious diseases. *Siam Rev.* **42,** 599–653 (2000).

5. Boily, M.-C. *et al.* Heterosexual risk of HIV-1 infection per sexual act: systematic review and meta-analysis of observational studies. *Lancet Infect. Dis.* **9,** 118–29 (2009).

6. Baggaley, R. F., White, R. G., Hollingsworth, T. D. & Boily, M.-C. Heterosexual HIV-1 infectiousness and antiretroviral use: systematic review of prospective studies of discordant couples. *Epidemiology* **24,** 110–21 (2013).

7. Bellan, S. E., Dushoff, J., Galvani, A. P. & Meyers, L. A. Reassessment of HIV-1 Acute Phase Infectivity: Accounting for Heterogeneity and Study Design with Simulated Cohorts. *PLoS Med.* **12,** e1001801 (2015).

8. Hollingsworth, T. D., Anderson, R. M. & Fraser, C. HIV-1 transmission, by stage of infection. *J. Infect. Dis.* **198,** 687–93 (2008).

9. Fiebig, E. W. *et al.* Dynamics of HIV viremia and antibody seroconversion in plasma donors: implications for diagnosis and staging of primary HIV infection. *AIDS* **17,** 1871–9 (2003).

10. Chemaitelly, H., Awad, S. F. & Abu-Raddad, L. J. The risk of HIV transmission within HIV-1 sero-discordant couples appears to vary across sub-Saharan Africa. *Epidemics* **6,** 1–9 (2014).

**C. Supplemental Tables**

**Table S1. Summary of Demographic & Health Surveys data sets and grouping for analysis**.

| group | surveys |
| --- | --- |
| Burundi | Burundi 2010-11 |
| Congo | Congo 2009 |
| DRC | Democratic Republic of Congo 2007 |
| Ethiopia | Ethiopia 2005, Ethiopia 2011 |
| Gabon | Gabon 2012 |
| Kenya | Kenya 2003, Kenya 2008-09 |
| Lesotho | Lesotho 2004-05, Lesotho 2009-10 |
| Malawi | Malawi 2010 |
| Mozambique | Mozambique 2009 |
| Rwanda | Rwanda 2005, Rwanda 2010-11 |
| Swaziland | Swaziland 2006-07 |
| Tanzania | Tanzania 2004, Tanzania 2007-08, Tanzania 2012 |
| Uganda | Uganda 2011, Uganda 2004-05 |
| West Africa (WA) | Cote d’Ivoire 2005-06, Burkina Faso 2003-04, Burkina Faso 2010-11, Cameroon 2004, Cameroon 2011, Ghana 2003, Guinea 2005, Liberia 2007, Mali 2006-07, Niger 2006, Senegal 2005, Senegal 2010-11,  Sierra Leone 2008 |
| Zambia | Zambia 2007 |
| Zimbabwe | Zimbabwe 2005-06, Zimbabwe 2010-11 |

**Table S2. Summary of Demographic & Health Surveys data analyzed and inclusion criteria**. Total number of couples analyzed and the number (%) of couples excluded due to ≥1 partner missing HIV serostatus, polygamy, insufficient data to determine partnership duration, or inconsistencies in partnership duration, age at sexual debut, or age at partnership formation. The portion to the right of the vertical line shows the distribution of serostatuses amongst couples as well as the proportion of analyzed couples in which both individuals were in their first partnership.

**Inclusion Criteria Couples Analyzed**

| data set | couples | no serostatus | polygamous | missing data | data inconsistent | couples analyzed | first partnership | M-F- | M+F- | M-F+ | M+F+ |
| --- | --- | --- | --- | --- | --- | --- | --- | --- | --- | --- | --- |
| Burundi | 2023 | 90 (4.4%) | 63 (3.1%) | 315 (15.6%) | 372 (18.4%) | 1413 (69.8%) | 1171 (82.9%) | 1377 (97.5%) | 7 (0.5%) | 10 (0.7%) | 19 (1.3%) |
| Congo | 2520 | 93 (3.7%) | 259 (10.3%) | 509 (20.2%) | 557 (22.1%) | 1401 (55.6%) | 824 (58.8%) | 1333 (95.1%) | 27 (1.9%) | 28 (2%) | 13 (0.9%) |
| DRC | 2373 | 228 (9.6%) | 648 (27.3%) | 470 (19.8%) | 517 (21.8%) | 1074 (45.3%) | 778 (72.4%) | 1052 (98%) | 11 (1%) | 9 (0.8%) | 2 (0.2%) |
| Ethiopia | 9713 | 1050 (10.8%) | 732 (7.5%) | 2287 (23.5%) | 2250 (23.2%) | 4917 (50.6%) | 2597 (52.8%) | 4824 (98.1%) | 33 (0.7%) | 25 (0.5%) | 35 (0.7%) |
| Gabon | 1946 | 65 (3.3%) | 226 (11.6%) | 497 (25.5%) | 398 (20.5%) | 1020 (52.4%) | 477 (46.8%) | 939 (92.1%) | 23 (2.3%) | 43 (4.2%) | 15 (1.5%) |
| Kenya | 2861 | 550 (19.2%) | 308 (10.8%) | 311 (10.9%) | 698 (24.4%) | 1484 (51.9%) | 1158 (78%) | 1358 (91.5%) | 35 (2.4%) | 43 (2.9%) | 48 (3.2%) |
| Lesotho | 1640 | 265 (16.2%) | 55 (3.4%) | 190 (11.6%) | 426 (26%) | 968 (59%) | 895 (92.5%) | 647 (66.8%) | 104 (10.7%) | 59 (6.1%) | 158 (16.3%) |
| Malawi | 3764 | 424 (11.3%) | 371 (9.9%) | 842 (22.4%) | 732 (19.4%) | 2017 (53.6%) | 1441 (71.4%) | 1797 (89.1%) | 91 (4.5%) | 50 (2.5%) | 79 (3.9%) |
| Mozambi | 2831 | 337 (11.9%) | 393 (13.9%) | 574 (20.3%) | 728 (25.7%) | 1260 (44.5%) | 841 (66.7%) | 1047 (83.1%) | 80 (6.3%) | 66 (5.2%) | 67 (5.3%) |
| Rwanda | 5012 | 64 (1.3%) | 192 (3.8%) | 532 (10.6%) | 525 (10.5%) | 4006 (79.9%) | 3306 (82.5%) | 3837 (95.8%) | 51 (1.3%) | 31 (0.8%) | 87 (2.2%) |
| Swaziland | 802 | 143 (17.8%) | 56 (7%) | 132 (16.5%) | 248 (30.9%) | 387 (48.3%) | 234 (60.5%) | 226 (58.4%) | 30 (7.8%) | 32 (8.3%) | 99 (25.6%) |
| Tanzania | 9836 | 1385 (14.1%) | 1415 (14.4%) | 1346 (13.7%) | 1470 (14.9%) | 5472 (55.6%) | 3947 (72.1%) | 5131 (93.8%) | 138 (2.5%) | 107 (2%) | 96 (1.8%) |
| Uganda | 9344 | 506 (5.4%) | 2213 (23.7%) | 2807 (30%) | 2475 (26.5%) | 3434 (36.8%) | 2056 (59.9%) | 3153 (91.8%) | 96 (2.8%) | 82 (2.4%) | 103 (3%) |
| WA | 30805 | 2852 (9.3%) | 10070 (32.7%) | 6062 (19.7%) | 7232 (23.5%) | 12124 (39.4%) | 7852 (64.8%) | 11692 (96.4%) | 167 (1.4%) | 174 (1.4%) | 91 (0.8%) |
| Zambia | 3129 | 829 (26.5%) | 293 (9.4%) | 669 (21.4%) | 614 (19.6%) | 1426 (45.6%) | 1029 (72.2%) | 1177 (82.5%) | 94 (6.6%) | 55 (3.9%) | 100 (7%) |
| Zimbabwe | 5567 | 1352 (24.3%) | 504 (9.1%) | 862 (15.5%) | 1320 (23.7%) | 2638 (47.4%) | 1949 (73.9%) | 2081 (78.9%) | 165 (6.3%) | 107 (4.1%) | 285 (10.8%) |

**Table S3. Estimated contact coefficients along with SDP and prevalence by country.** DHS prevalence and SDP show the HIV prevalence and serodiscordant proportion in the couples population as sampled by DHS and analyzed in this study. Peak prevalence shows the estimated peak adult HIV prevalence from UNAIDS models. Estimated contact coefficients are shown with 95% credible intervals for pre-couple (men and women separate) and extra-couple (geometric average across genders is shown because they were similar; this is what was fit in the regression sin the main analysis.

|  | DHS prevalence | peak prevalence | SDP | *c*_pre,male_ | *c*_pre,female_ | *c*_extra,average_ |
| --- | --- | --- | --- | --- | --- | --- |
| Burundi | 0.019 | 0.045 | 0.47 | 1.4 (0.16, 4.2) | 7.3 (2.6, 17) | 0.7 (0.35, 1.3) |
| Congo | 0.029 | 0.048 | 0.81 | 1.4 (0.32, 3.8) | 6.2 (2.8, 14) | 1.9 (0.92, 3.9) |
| DRC | 0.011 | 0.014 | 0.91 | 5.6 (0.24, 40) | 6 (0.23, 48) | 5.6 (1.7, 30) |
| Ethiopia | 0.013 | 0.032 | 0.62 | 2.8 (1.4, 5.1) | 11 (5.9, 19) | 0.43 (0.21, 0.73) |
| Gabon | 0.047 | 0.055 | 0.81 | 2.4 (0.99, 5.8) | 16 (8, 35) | 2.1 (1, 4.4) |
| Kenya | 0.059 | 0.091 | 0.62 | 1.4 (0.71, 2.4) | 5.5 (3.3, 9.1) | 0.59 (0.39, 0.92) |
| Lesotho | 0.25 | 0.23 | 0.51 | 1.4 (0.88, 2.2) | 3.4 (2.1, 5.3) | 1.3 (1, 1.7) |
| Malawi | 0.084 | 0.15 | 0.61 | 1 (0.52, 1.7) | 4.7 (2.9, 7.4) | 0.86 (0.61, 1.2) |
| Mozambique | 0.11 | 0.094 | 0.69 | 3.6 (2.1, 6.1) | 12 (7.9, 20) | 1.9 (1.4, 2.8) |
| Rwanda | 0.032 | 0.059 | 0.49 | 1.6 (0.9, 2.5) | 3.7 (2.1, 5.9) | 0.54 (0.38, 0.76) |
| Swaziland | 0.34 | 0.23 | 0.39 | 2.3 (1.4, 3.9) | 5.4 (3.5, 8.5) | 0.6 (0.37, 0.95) |
| Tanzania | 0.04 | 0.073 | 0.72 | 1.8 (1.2, 2.6) | 5.7 (4, 8.3) | 0.94 (0.71, 1.3) |
| Uganda | 0.056 | 0.12 | 0.63 | 1.6 (1.1, 2.3) | 3.8 (2.6, 5.6) | 0.75 (0.57, 1) |
| WA | 0.021 | 0.031 | 0.78 | 2.8 (1.1, 4.2) | 10 (4.4, 14) | 2.3 (1, 3) |
| Zambia | 0.12 | 0.15 | 0.6 | 1.9 (1.2, 2.8) | 3.9 (2.6, 5.7) | 0.7 (0.51, 0.98) |
| Zimbabwe | 0.16 | 0.26 | 0.49 | 1.2 (0.85, 1.6) | 4.4 (3.3, 5.9) | 0.68 (0.56, 0.83) |

**Table S4.** **Model selection via AICc.** Model selection table using AICc to choose amongst candidate multivariate models of log-odds prevalence for an acute phase to chronic phase relative hazard ($\alpha)$ of 7, and also showing the robustness of the model selection algorithm for the range of $\alpha=1-50$.

|  | $\boldsymbol{\alpha=7}$ | | | | | $\boldsymbol{\alpha=1}$ | $\boldsymbol{\alpha=25}$ | $\boldsymbol{\alpha=50}$ |
| --- | --- | --- | --- | --- | --- | --- | --- | --- |
| model | df | AICc | $\boldsymbol{\Delta}$AICc | AICc weight | log likelihood | $\boldsymbol{\Delta}$AICc | $\boldsymbol{\Delta}$AICc | $\boldsymbol{\Delta}$AICc |
| HIV transmission rate | 3 | 38 | 0 | 0.797 | -15 | 0 | 0 | 0 |
| female pre-couple contact coefficient | 3 | 42.3 | 4.27 | 0.0943 | -17.1 | 3.67 | 4.32 | 3.74 |
| male pre-couple contact coefficient | 3 | 43.2 | 5.19 | 0.0595 | -17.6 | 3.83 | 5.85 | 5.53 |
| full multivariate model (all 4 predictors) | 6 | 43.9 | 5.9 | 0.0417 | -11.3 | 5.8 | 6.25 | 6.74 |
| extra-couple contact coefficient | 3 | 47.4 | 9.39 | 0.00729 | -19.7 | 8.87 | 9.15 | 7.81 |

**Table S5. Estimated HIV transmission rates.** Estimated transmission rates experienced by men (M), women (F), and their geometric average (G) along with 95% credible intervals estimated by country and across a range of assumptions for the relative hazard of the acute to chronic phase.

|  |  | acute to chronic phase relative hazard assumed during model fitting | | | | | | | |
| --- | --- | --- | --- | --- | --- | --- | --- | --- | --- |
|  |  | 1 | 5 | 7 | 10 | 25 | 30 | 40 | 50 |
| Burundi | M | 15 (6.7, 28) | 14 (6.3, 26) | 13 (6.1, 25) | 13 (5.9, 23) | 10 (4.9, 18) | 9.6 (4.7, 17) | 8.7 (4.1, 15) | 8.1 (3.9, 14) |
|  | F | 19 (9.1, 37) | 17 (8.5, 32) | 16 (8.1, 30) | 15 (7.5, 28) | 12 (6, 20) | 11 (5.6, 18) | 9.4 (4.8, 16) | 8.4 (4.3, 14) |
|  | G | 17 (9.2, 29) | 15 (8.5, 25) | 15 (8.3, 24) | 14 (8, 22) | 11 (6.5, 17) | 10 (6.2, 16) | 9 (5.3, 14) | 8.1 (5, 12) |
| Congo | M | 4.7 (2.2, 8.9) | 4.5 (2.1, 8.4) | 4.3 (2, 8) | 4.1 (1.8, 7.9) | 3.5 (1.6, 6.6) | 3.3 (1.4, 6.3) | 3 (1.3, 5.7) | 2.8 (1.3, 5.2) |
|  | F | 4.1 (1.7, 8.2) | 3.7 (1.6, 7.4) | 3.6 (1.5, 7.2) | 3.4 (1.5, 6.9) | 2.8 (1.2, 5.5) | 2.7 (1.2, 5.2) | 2.5 (1.1, 4.7) | 2.2 (0.99, 4.3) |
|  | G | 4.4 (2.3, 7.4) | 4.1 (2.1, 7) | 3.9 (2, 6.6) | 3.8 (1.9, 6.4) | 3.1 (1.6, 5.3) | 3 (1.6, 5) | 2.7 (1.4, 4.5) | 2.5 (1.3, 4.2) |
| DRC | M | 2.7 (0.49, 9.1) | 2.5 (0.46, 8.3) | 2.4 (0.41, 7.9) | 2.3 (0.44, 8.1) | 1.7 (0.32, 5.6) | 1.6 (0.28, 4.9) | 1.4 (0.26, 4.5) | 1.2 (0.23, 4) |
|  | F | 2.4 (0.42, 7.9) | 2.2 (0.39, 7.1) | 2.1 (0.36, 7) | 2 (0.39, 6.6) | 1.5 (0.29, 4.7) | 1.4 (0.25, 4.3) | 1.2 (0.23, 3.9) | 1.1 (0.2, 3.5) |
|  | G | 2.6 (0.49, 7.9) | 2.3 (0.45, 7.1) | 2.3 (0.42, 6.6) | 2.2 (0.44, 6.7) | 1.6 (0.31, 4.7) | 1.6 (0.28, 4.1) | 1.3 (0.26, 3.8) | 1.2 (0.23, 3.4) |
| Ethiopia | M | 7.3 (3.9, 12) | 7 (3.7, 11) | 6.8 (3.7, 11) | 6.7 (3.6, 11) | 6 (3.1, 9.9) | 5.8 (3.1, 9.6) | 5.5 (2.9, 8.9) | 5.2 (2.7, 8.3) |
|  | F | 8.1 (4.7, 13) | 7.6 (4.5, 12) | 7.4 (4.4, 11) | 7.2 (4.2, 11) | 6.1 (3.7, 9.3) | 5.7 (3.4, 8.8) | 5.3 (3.2, 8) | 4.9 (3, 7.5) |
|  | G | 7.6 (5.2, 11) | 7.2 (4.9, 10) | 7.1 (4.9, 9.9) | 6.9 (4.7, 9.7) | 6 (4.1, 8.4) | 5.7 (3.9, 8) | 5.3 (3.7, 7.3) | 5 (3.4, 6.9) |
| Gabon | M | 5.1 (2.4, 9.2) | 4.7 (2.2, 8.5) | 4.7 (2.2, 8.4) | 4.4 (2.1, 8) | 3.7 (1.8, 6.6) | 3.5 (1.6, 6.3) | 3.2 (1.5, 5.7) | 2.9 (1.3, 5.2) |
|  | F | 3.8 (1.5, 8.1) | 3.6 (1.4, 7.9) | 3.5 (1.4, 7.6) | 3.3 (1.3, 7.2) | 2.8 (1.1, 6) | 2.7 (1.1, 5.7) | 2.5 (0.96, 5.3) | 2.2 (0.88, 4.7) |
|  | G | 4.4 (2.2, 7.5) | 4.1 (2.1, 7.2) | 4 (2, 7) | 3.8 (1.9, 6.6) | 3.2 (1.6, 5.6) | 3.1 (1.6, 5.3) | 2.8 (1.4, 4.8) | 2.5 (1.3, 4.4) |
| Kenya | M | 11 (5.8, 17) | 10 (5.7, 16) | 9.8 (5.6, 15) | 9.3 (5.2, 14) | 7.8 (4.4, 12) | 7.4 (4.1, 11) | 6.7 (3.7, 10) | 6.1 (3.5, 9.3) |
|  | F | 11 (6, 18) | 9.9 (5.3, 16) | 9.5 (5.1, 16) | 9 (4.8, 15) | 7 (3.7, 11) | 6.6 (3.6, 11) | 5.8 (3.1, 9.2) | 5.2 (2.7, 8.4) |
|  | G | 11 (7.4, 15) | 9.8 (6.8, 14) | 9.6 (6.7, 13) | 9.1 (6.4, 12) | 7.3 (5.1, 9.9) | 6.9 (4.8, 9.3) | 6.1 (4.3, 8.3) | 5.6 (4, 7.5) |
| Lesotho | M | 15 (7.9, 25) | 13 (6.7, 22) | 13 (6.4, 21) | 12 (5.9, 19) | 8.8 (4.4, 14) | 8.2 (4, 13) | 7.1 (3.6, 11) | 6.3 (3.3, 9.9) |
|  | F | 17 (11, 24) | 15 (9.3, 20) | 14 (8.6, 19) | 13 (8.1, 17) | 8.9 (5.5, 12) | 8 (5, 11) | 6.9 (4.2, 9.3) | 5.9 (3.5, 8.1) |
|  | G | 16 (12, 20) | 14 (10, 17) | 13 (9.8, 16) | 12 (9.1, 15) | 8.7 (6.6, 11) | 8 (6, 9.7) | 6.8 (5.2, 8.3) | 6 (4.7, 7.4) |
| Malawi | M | 9.1 (5, 15) | 8.4 (4.8, 13) | 8.3 (4.7, 13) | 8 (4.4, 12) | 6.5 (3.6, 10) | 6.2 (3.4, 9.5) | 5.6 (3, 9.6) | 5.2 (2.9, 8.6) |
|  | F | 7 (4.2, 10) | 6.4 (3.8, 9.5) | 6.1 (3.6, 8.9) | 5.7 (3.5, 8.4) | 4.5 (2.7, 6.5) | 4.2 (2.4, 6.1) | 3.7 (2.1, 5.4) | 3.3 (1.9, 4.8) |
|  | G | 7.9 (5.9, 10) | 7.3 (5.4, 9.4) | 7 (5.2, 9) | 6.6 (5, 8.6) | 5.3 (4, 6.8) | 5 (3.7, 6.4) | 4.4 (3.3, 6.1) | 4.1 (3, 5.4) |
| Mozambique | M | 11 (6.7, 17) | 10 (6, 15) | 9.6 (5.7, 14) | 8.9 (5.3, 13) | 6.9 (4.1, 10) | 6.3 (3.7, 9.5) | 5.5 (3.2, 8.2) | 4.9 (2.8, 7.3) |
|  | F | 6.6 (3.5, 11) | 5.8 (3.1, 9.6) | 5.5 (3, 9.1) | 5.1 (2.7, 8.4) | 3.8 (2.1, 6.2) | 3.5 (1.9, 5.8) | 3 (1.7, 5) | 2.8 (1.5, 4.4) |
|  | G | 8.5 (6, 11) | 7.6 (5.4, 10) | 7.2 (5.1, 9.7) | 6.7 (4.8, 9.1) | 5.1 (3.6, 6.8) | 4.7 (3.3, 6.3) | 4 (2.9, 5.4) | 3.6 (2.6, 4.8) |
| Rwanda | M | 16 (9.3, 27) | 15 (8.5, 24) | 15 (8.3, 23) | 14 (7.8, 21) | 11 (6.5, 17) | 11 (6.1, 16) | 9.5 (5.6, 14) | 8.8 (5.2, 13) |
|  | F | 16 (11, 22) | 14 (9.9, 20) | 14 (9.7, 19) | 13 (9, 18) | 10 (7.1, 14) | 9.6 (6.8, 13) | 8.4 (5.8, 11) | 7.5 (5.2, 10) |
|  | G | 16 (12, 21) | 15 (11, 19) | 14 (10, 18) | 13 (9.9, 17) | 11 (8, 13) | 10 (7.7, 12) | 8.9 (6.9, 11) | 8.1 (6.3, 10) |
| Swaziland | M | 24 (13, 39) | 21 (12, 34) | 20 (11, 32) | 19 (11, 30) | 15 (8.6, 23) | 14 (8.2, 21) | 12 (7.1, 19) | 11 (6.4, 18) |
|  | F | 21 (12, 36) | 19 (10, 31) | 17 (9.6, 29) | 16 (8.8, 26) | 11 (6.3, 18) | 10 (5.9, 17) | 9 (5, 14) | 7.9 (4.5, 12) |
|  | G | 22 (15, 32) | 20 (14, 27) | 19 (13, 26) | 17 (12, 24) | 13 (9.1, 17) | 12 (8.4, 16) | 10 (7.3, 14) | 9.4 (6.6, 13) |
| Tanzania | M | 10 (6.7, 15) | 9.5 (6.2, 13) | 9.2 (6, 13) | 8.8 (5.7, 12) | 7 (4.6, 9.6) | 6.7 (4.3, 9.1) | 5.9 (3.8, 8.2) | 5.4 (3.5, 8) |
|  | F | 5.3 (2.9, 8.4) | 4.9 (2.8, 7.6) | 4.7 (2.6, 7.4) | 4.4 (2.5, 6.9) | 3.5 (1.9, 5.5) | 3.3 (1.8, 5.2) | 2.9 (1.6, 4.6) | 2.6 (1.4, 4.1) |
|  | G | 7.4 (5.5, 9.3) | 6.7 (5.2, 8.5) | 6.5 (5, 8.2) | 6.2 (4.7, 7.7) | 4.9 (3.8, 6.1) | 4.6 (3.5, 5.8) | 4.1 (3.2, 5.1) | 3.7 (2.9, 4.9) |
| Uganda | M | 13 (8.2, 18) | 12 (7.6, 17) | 11 (7.1, 16) | 10 (6.8, 15) | 8.4 (5.5, 12) | 7.9 (5.2, 11) | 7 (4.5, 9.6) | 6.4 (4.1, 8.7) |
|  | F | 8.5 (5.1, 13) | 7.7 (4.5, 12) | 7.5 (4.5, 11) | 7 (4.2, 11) | 5.6 (3.4, 8.4) | 5.3 (3.1, 7.9) | 4.7 (2.7, 7.1) | 4.2 (2.4, 6.4) |
|  | G | 10 (8, 13) | 9.4 (7.3, 12) | 9 (7, 11) | 8.5 (6.7, 11) | 6.8 (5.3, 8.4) | 6.4 (5, 7.9) | 5.7 (4.5, 7) | 5.1 (4, 6.3) |
| WA | M | 4.9 (2.8, 7.5) | 4.6 (2.5, 7.3) | 4.5 (2.5, 7.3) | 4.3 (2.5, 6.6) | 3.5 (2, 18) | 3.3 (1.8, 21) | 3 (1.7, 20) | 2.8 (1.6, 43) |
|  | F | 6.1 (3.8, 8.8) | 5.5 (3.3, 8) | 5.2 (3.1, 7.4) | 4.8 (2.9, 6.9) | 3.5 (2, 5.1) | 3.3 (1.9, 4.8) | 2.8 (1.6, 4.1) | 2.4 (1.4, 3.6) |
|  | G | 5.4 (4.2, 6.8) | 5 (3.9, 6.4) | 4.8 (3.6, 6.3) | 4.5 (3.5, 5.6) | 3.5 (2.7, 8) | 3.2 (2.5, 8.5) | 2.8 (2.1, 7.6) | 2.6 (2, 9.7) |
| Zambia | M | 13 (7.6, 20) | 12 (7.1, 19) | 12 (6.8, 18) | 12 (6.6, 17) | 9.6 (5.6, 14) | 9.1 (5.3, 13) | 8.2 (4.7, 12) | 7.4 (4.3, 11) |
|  | F | 9.2 (5.6, 13) | 8.3 (5.1, 12) | 8 (4.7, 12) | 7.5 (4.5, 11) | 5.9 (3.5, 8.5) | 5.5 (3.2, 8.1) | 4.9 (2.9, 7) | 4.4 (2.6, 6.4) |
|  | G | 11 (8.3, 14) | 10 (7.6, 13) | 9.7 (7.3, 12) | 9.2 (7, 12) | 7.4 (5.7, 9.2) | 6.9 (5.4, 8.8) | 6.2 (4.8, 7.8) | 5.6 (4.3, 7) |
| Zimbabwe | M | 16 (11, 22) | 15 (10, 20) | 15 (9.8, 20) | 14 (9.5, 19) | 11 (8, 15) | 11 (7.7, 14) | 9.9 (7, 13) | 9 (6.3, 12) |
|  | F | 13 (9.2, 17) | 12 (8.3, 15) | 11 (7.7, 15) | 10 (7, 13) | 7.5 (5, 10) | 6.9 (4.5, 9.3) | 6 (3.8, 8.2) | 5.2 (3.3, 7.2) |
|  | G | 14 (12, 17) | 13 (11, 15) | 13 (11, 15) | 12 (10, 14) | 9.2 (7.9, 11) | 8.6 (7.4, 9.8) | 7.6 (6.5, 8.9) | 6.8 (5.7, 7.8) |

**Table S6.** **Comparison of HIV-1 transmission rate estimates with Chemaitelly et al. (2014).** Point estimates are given only; for confidence intervals see Table 2 in the main text of this paper and Table S1 of Chemaitelly et al. (2014).

|  | Bellan et al. | | Chemaitelly et al. |
| --- | --- | --- | --- |
| Country | **male** | **female** | **gender-average** |
| Burundi | 13 | 16 | 30 |
| Congo | 4.3 | 3.6 | 4.4 |
| DRC | 2.4 | 2.1 | 6.5 |
| Ethiopia | 6.8 | 7.4 | 17 |
| Gabon | 4.7 | 3.5 | not analyzed |
| Kenya | 9.8 | 9.5 | 15 |
| Lesotho | 13 | 14 | 26 |
| Malawi | 8.3 | 6.1 | 20 |
| Mozambique | 9.6 | 5.5 | 12 |
| Rwanda | 15 | 14 | 33 |
| Swaziland | 20 | 17 | 41 |
| Tanzania | 9.2 | 4.7 | 10 |
| Uganda | 11 | 7.5 | not analyzed |
| West Africa* | 4.5 | 5.2 | 11, 4.5, 7.7, 4.4, 7.7, 6.8, 9.6, 7.2, 13.8 |
| Zambia | 12 | 8 | 19.5 |
| Zimbabwe | 15 | 11 | 27 |

*Because we fit our model to individual-level data in this paper, the HIV-positive sample sizes in West African countries, which all have relatively low HIV prevalence, were too small for our model fits to converge. We thus pooled these countries. Chemaitelly et al. in contrast fit their model to population summary statistics and were thus able to fit each country’s data set. We thus listed their estimates of transmission rates for Senegal, Niger, Mali, Liberia, Burkina Faso, Guinea, Ghana, Cote d’Ivoire and Cameroon (in order of appearance above), which we included in our pooled West African analysis.

**D. Supplemental Figures**

**Figure S1**. **Simulated relationship histories and serodiscordant proportions.**

Panels A-C have the same time axis labeled at the bottom of panel C. (A) An illustrative schematic diagram of the HIV transmission model with an example Zambian couple’s relationship history. Each partner (black lines) can be infected pre-couple formation beginning from the month of their sexual debut until the month the couple is formed (gray arrows and hazards, $\lambda$). After couple formation, an individual can be infected by their partner if their partner is positive (blue arrows and hazards, $\lambda$), or from extra-couple intercourse (red arrows and hazards, $\lambda$). Pre-couple and extra-couple hazards vary with the HIV infectious prevalence (prevalence of infected individuals not on ART) in the opposite sex ^2^. (B) The relationship histories of ten example simulated couples are shown along with discrete infection (triangles pointing down and up show infections of men and women, respectively). Relationship lines terminate when one of the partners ages out of the population sampled by the Demographic and Health Surveys (at 50 and 60 years old for women and men, respectively) or when a partner dies of AIDS-related mortality. (C) The serodiscordant proportion (SDP; i.e., proportion of couples with infected individuals that are serodiscordant) over time for the 10 couples in the top panel as well as for a 100,000 simulated couples (population size used in the analyses in Figures 3-4).


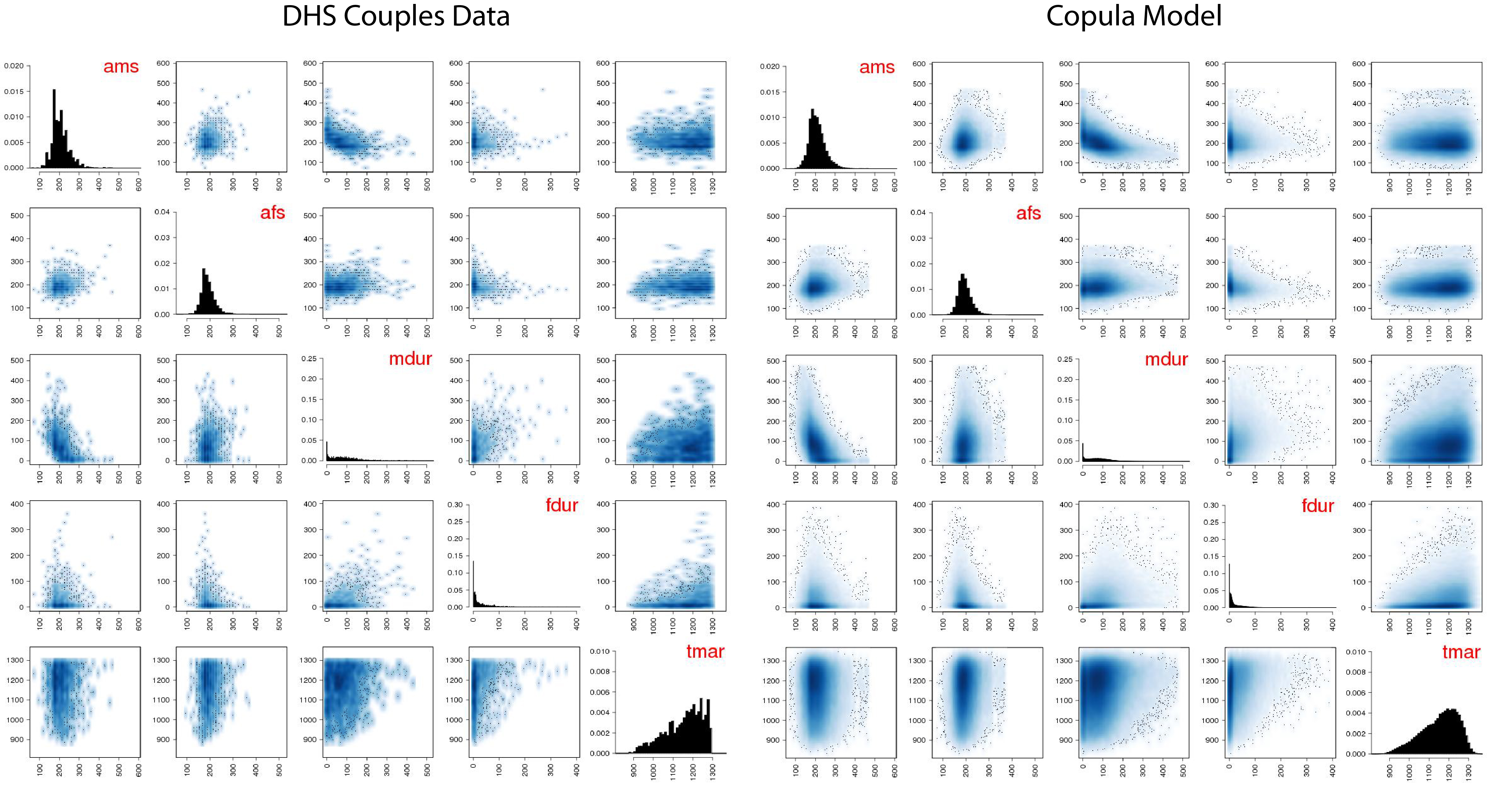


**Figure S2. Survival-inflated copula model of couple relationship histories for Zambia.** Left panel shows pairwise density plots of the five variables that comprise each couple’s relationship history from the Zambian DHS: age at male and female sexual debut (ams and afs), male and female duration of sexual activity prior to couple formation (mdur and fdur), and date of couple formation (tmar). All values are shown in months or months since 1900 (tmar). The right panel shows multivariate copula model fit to these data to simulate couples representative of the multivariate correlated relationship between these variables in Zambia. Note that we simulated the first four variables conditional on the latte (tmar), where couple cohorts (defined by date of couple formation) of equal size were used for the period simulated.


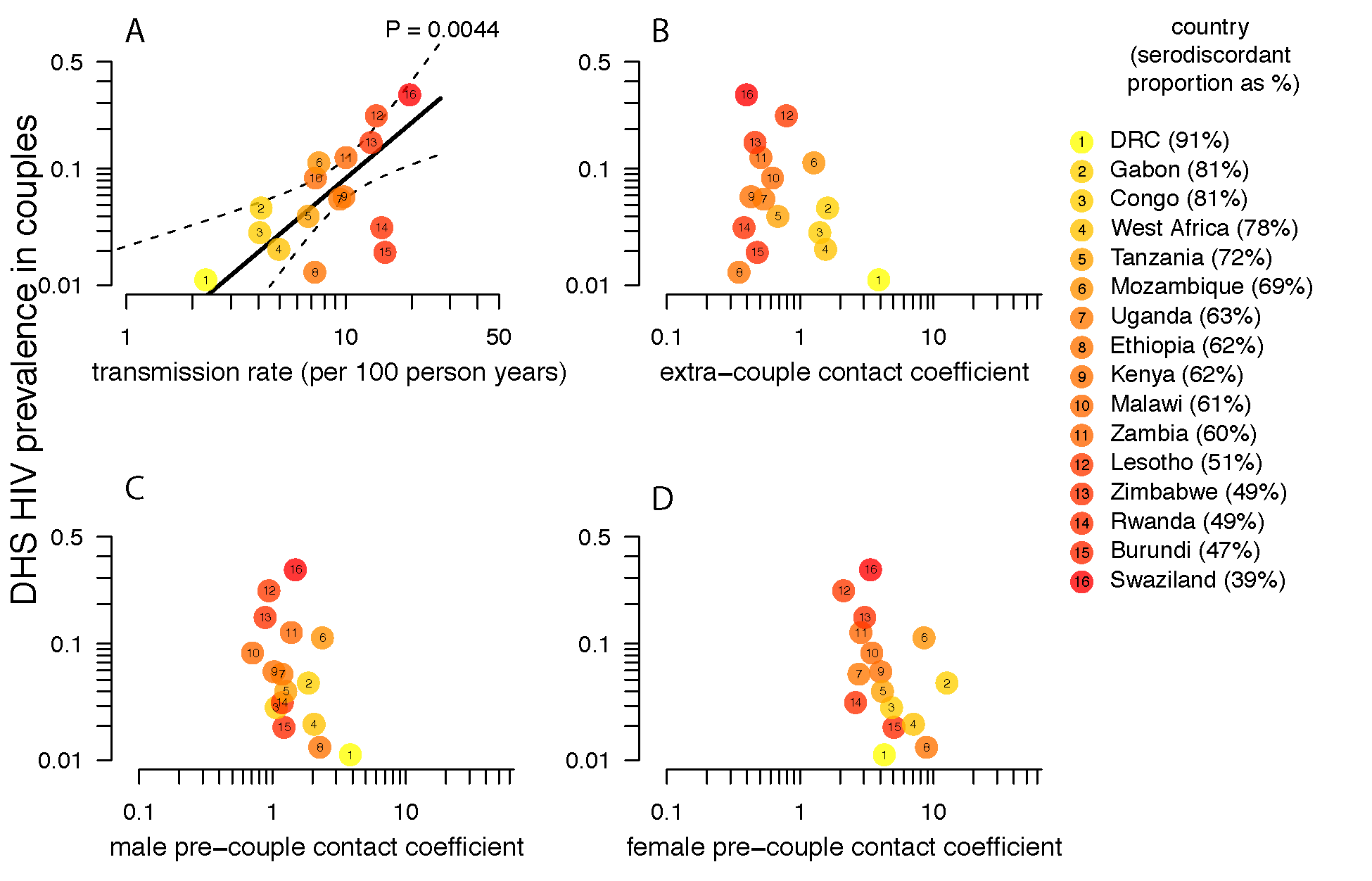


**Figure S3.** Figure 2 from the main text replicated but with HIV prevalence amongst the DHS data (averaged for each country data set) analyzed used as the meta-regression dependent variable, instead of UNAIDS-estimated peak prevalence. Results are qualitatively similar for both outcomes.

**Figure S4. Sensitivity of estimated transmission coefficients to assumed acute to chronic phase relative hazard.** Estimated pre-couple ($\beta_{M,b}$, $\beta_{F,b}$; recall that we define these as $\beta_{M,b}=\beta_{M,p}c_{M,b}$ where ${\beta_{M,p}=\beta}_{M}^{*}$ is the intrinsic transmission rate to males and similarly for females) and extra-couple contact coefficients ($\beta_{M,e}$, $\beta_{F,e}$) are remarkably stable across a wide range of assumed values for the increased infectiousness of the acute phase. The within-couple transmission coefficient ($\beta_{M,p}$, $\beta_{F,p}$; the intrinsic transmission rate during the chronic phase)—necessarily declines with greater assumed acute phase infectiousness to result in the same total experienced infectiousness over the natural history of disease. All lines show posterior medians. Burundi’s estimates appear to be sensitive to the acute phase relative hazard; this is entirely due to the small number of infected individuals sampled in Burundi’s DHS and is also reflected in wide credible intervals around these values (not shown here; see Table S3).

 **Figure S5**. **Counter-factual versus observed transmission patterns.** Panel I) is a schematic diagram of the patterns found in Figure 4 of the main text. County-to-country variation in the transmission rate accounts for variation in peak HIV prevalence, while contact coefficients do not. Because the former is closely tied to the serodiscordant proportion (SDP), high prevalence countries also have small SDPs. If, in we consider the counter-factual scenario which between-country differences in contact coefficients drove epidemic prevalence then we would have expected Figure 4 to look more like panel II. In this scenario, there is little variability in transmission rates and so the SDP’s are relatively similar between countries; estimated contact coefficients, in contrast, would be greater (for at least one of panels (B)-(D)) for countries with more severe epidemics (as measured by peak prevalence).


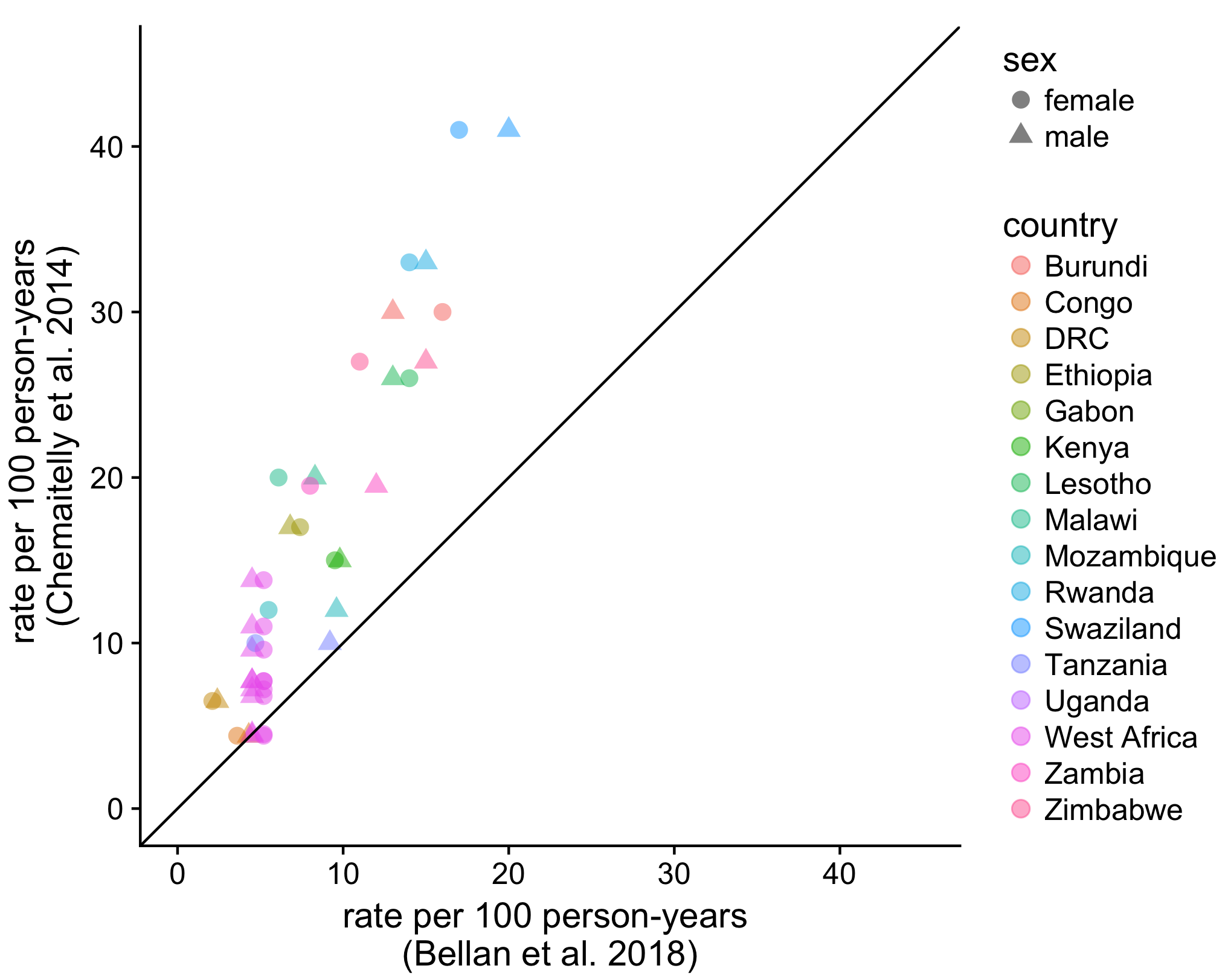


**Fig S6 Comparison of estimated transmission rates from this manuscript with those of Chemaitelly et al. 2014**^10^**.** Symbol indicates gender-specificity of transmission rate for estimates made in this manuscript (estimates from Chemaitelly were not gender-specific). Estimates from Chemaitelly et al.’s analysis were generally twice as large as those from our analysis, though country-ordering of transmission rates remains similar. We believe the discrepancy arises because we used individual-level data on relationships, which allowed us to better account for person-time at risk of infection to infection. In contrast, Chemaitelly et al.’s analysis reliance on population-level summary statistics means that only average couple duration, SDP, and other country-level statistics were used to estimate transmission.

**Figure S7**. **Serodiscordant proportion versus relationship duration and serodiscordant proportion by country.** This figure shows the relationship between the serodiscordant proportion and mean relationship duration of couples (both the raw mean and an AIDS-survival-inflated mean to account for under-sampling couples dissolved by the AIDS death of a partner) sampled in Demographic and Health Surveys for all analyzed couples (left) or for all analyzed couples in which both partners were in their first relationship (right). If variation in couple dissolution were the cause of variation in the serodiscordant proportion between countries, we would expect countries with the greatest dissolution rates (i.e., shortest mean relationship durations) to have the greatest serodiscordance proportion (particularly among couples in their first union). However, the trend is in the opposite direction and statistically insignificant for all analyses suggesting this explanation is unlikely.


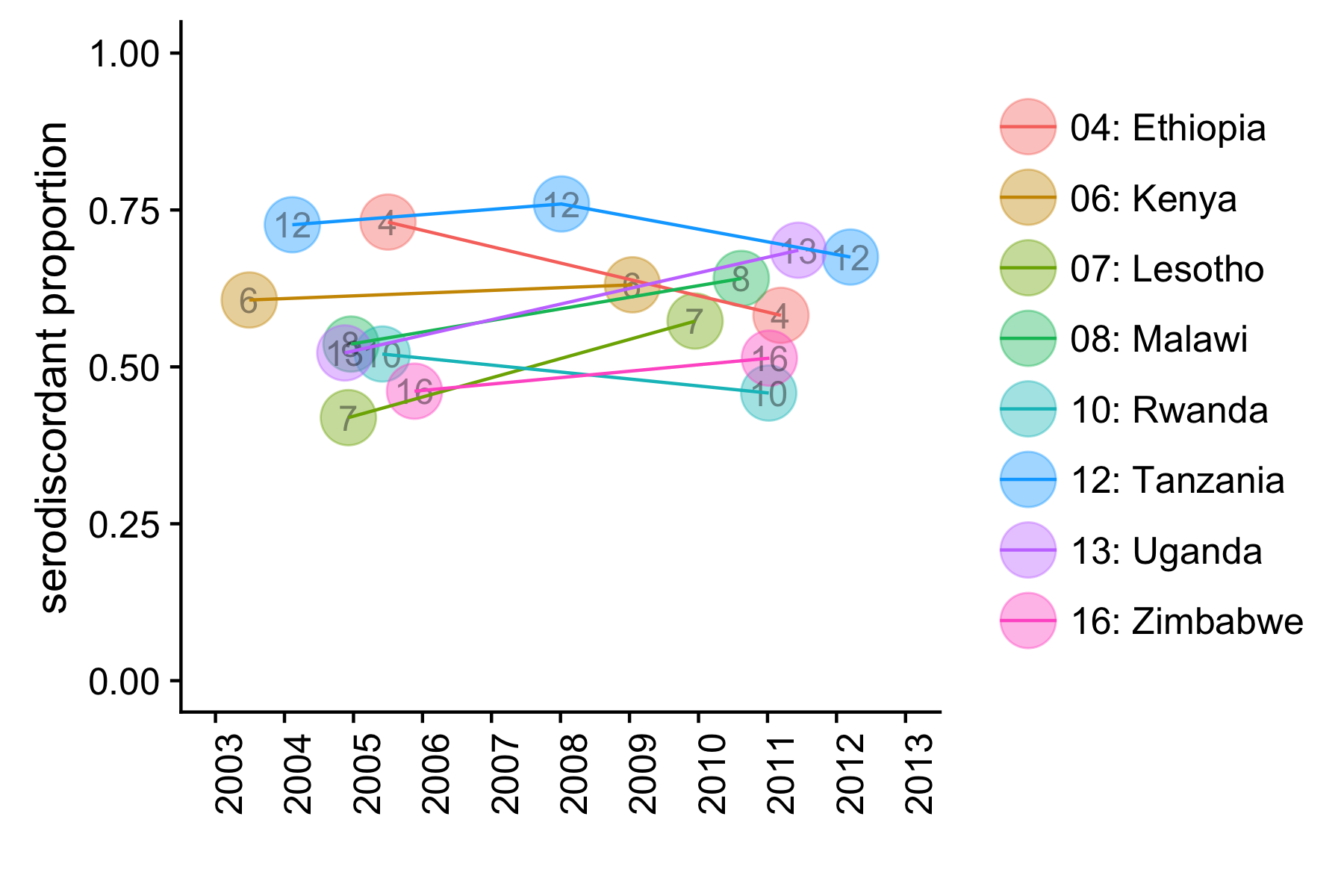


**Figure S8. Changes in serodiscordant proportion over time**. Shown for countries in which ≥1 survey was conducted during the study period (2003-2012).

1. Reprinted from The Lancet, 381:9877, p1561-1569, Bellan, Fiorella, Melesse, Getz, Williams and Dushoff, *Extra-couple HIV transmission in sub-Saharan Africa: a mathematical modelling study of survey data*, p1561-1569, 2013, with permission from Elsevier and The Lancet. [↑](#footnote-ref-1)
